# Gram-negative neonatal sepsis in low- and lower-middle-income countries and WHO empirical antibiotic recommendations: A systematic review and meta-analysis

**DOI:** 10.1371/journal.pmed.1003787

**Published:** 2021-09-28

**Authors:** Sophie C. H. Wen, Yukiko Ezure, Lauren Rolley, Geoff Spurling, Colleen L. Lau, Saba Riaz, David L. Paterson, Adam D. Irwin

**Affiliations:** 1 Centre for Clinical Research, University of Queensland, Brisbane, Queensland, Australia; 2 Infection Management Prevention Service, Queensland Children’s Hospital, Brisbane, Queensland, Australia; 3 School of Public Health, University of Queensland, Brisbane, Queensland, Australia; 4 Primary Care Clinical Unit, University of Queensland, Brisbane, Queensland, Australia; 5 Research School of Population Health, Australian National University, Canberra, Australian Capital Territory, Australia; 6 Institute of Microbiology and Molecular Genetics, University of the Punjab, Lahore, Pakistan; 7 Royal Brisbane and Women’s Hospital, Brisbane, Queensland, Australia; The Hospital for Sick Children, CANADA

## Abstract

**Background:**

Neonatal sepsis is a significant global health issue associated with marked regional disparities in mortality. Antimicrobial resistance (AMR) is a growing concern in Gram-negative organisms, which increasingly predominate in neonatal sepsis, and existing WHO empirical antibiotic recommendations may no longer be appropriate. Previous systematic reviews have been limited to specific low- and middle-income countries. We therefore completed a systematic review and meta-analysis of available data from all low- and lower-middle-income countries (LLMICs) since 2010, with a focus on regional differences in Gram-negative infections and AMR.

**Methods and findings:**

All studies published from 1 January 2010 to 21 April 2021 about microbiologically confirmed bloodstream infections or meningitis in neonates and AMR in LLMICs were assessed for eligibility. Small case series, studies with a small number of Gram-negative isolates (<10), and studies with a majority of isolates prior to 2010 were excluded. Main outcomes were pooled proportions of *Escherichia coli*, *Klebsiella*, *Enterobacter*, *Pseudomonas*, *Acinetobacter* and AMR. We included 88 studies (4 cohort studies, 3 randomised controlled studies, and 81 cross-sectional studies) comprising 10,458 Gram-negative isolates from 19 LLMICs. No studies were identified outside of Africa and Asia. The estimated pooled proportion of neonatal sepsis caused by Gram-negative organisms was 60% (95% CI 55% to 65%). *Klebsiella* spp. was the most common, with a pooled proportion of 38% of Gram-negative sepsis (95% CI 33% to 43%). Regional differences were observed, with higher proportions of *Acinetobacter* spp. in Asia and *Klebsiella* spp. in Africa. Resistance to aminoglycosides and third-generation cephalosporins ranged from 42% to 69% and from 59% to 84%, respectively. Study limitations include significant heterogeneity among included studies, exclusion of upper-middle-income countries, and potential sampling bias, with the majority of studies from tertiary hospital settings, which may overestimate the burden caused by Gram-negative bacteria.

**Conclusions:**

Gram-negative bacteria are an important cause of neonatal sepsis in LLMICs and are associated with significant rates of resistance to WHO-recommended first- and second-line empirical antibiotics. AMR surveillance should underpin region-specific empirical treatment recommendations. Meanwhile, a significant global commitment to accessible and effective antimicrobials for neonates is required.

## Abstract 2

Sophie Wen and co-workers report a meta-analysis on bacterial isolates and antimicrobial resistance in neonatal sepsis in low- and lower-middle-income countries.

## Author summary

**Why was this study done?:**

- Neonatal sepsis is a significant cause of childhood mortality with marked disparities across world regions and countries.
- Gram-negative bacteria are becoming increasingly predominant in neonatal sepsis, particularly in low- and middle-income countries, along with growing concerns of multidrug resistance.
- It is likely that the World Health Organization (WHO) empirical neonatal sepsis antibiotic recommendations are no longer adequate. We analysed data on neonatal sepsis from low- and lower-middle-income countries (LLMICs) to assess the burden of Gram-negative infections and associated antimicrobial resistance (AMR).

**What did the researchers do and find?:**

- We pooled data from 88 published studies from 19 LLMICs on neonatal sepsis (with a total of 10,458 Gram-negative isolates) and summarised the proportions of Gram-negative sepsis caused by *E*. *coli*, *Klebsiella*, *Enterobacter*, *Pseudomonas*, and *Acinetobacter*. We also examined the rates of AMR against key empirical antibiotics used in neonatal sepsis.
- Gram-negative bacteria accounted for 60% of neonatal sepsis in LLMICs. Regional variations in types of Gram-negative bacteria were observed, along with high rates of resistance against the WHO-recommended empirical antibiotics for neonatal sepsis.

**What do these findings mean?:**

- The WHO empirical antibiotic recommendations for neonatal sepsis are likely inadequate in many LLMICs.
- Robust AMR surveillance and reporting in LLMICs is necessary to develop region-specific empirical antibiotic recommendations for neonatal sepsis.

## Introduction

Neonatal sepsis is a major cause of mortality and morbidity, accounting for approximately 22% of global annual neonatal deaths [1]. Improvements in neonatal mortality over the last 30 years have occurred at a slower rate than those observed for post-neonatal mortality, and the neonatal period contributes greater than 40% of all mortality in children under 5 years of age. The Sustainable Development Goals (SDGs) target a reduction of neonatal mortality in all countries to less than 12 deaths per 1,000 live births by 2030 [1,2]. There is significant variation in the reported incidence of neonatal sepsis worldwide, with a paucity of data particularly from low-income countries. In high- and middle-income countries, it has been estimated that neonatal sepsis occurs in 2,200 neonates per 100,000 live births, equating to 3 million cases of neonatal sepsis annually, with a mortality rate of 11% to 19% [3]. The incidence of neonatal sepsis in middle-income countries has been reported to be up to 40 times higher than in high-income countries [3].

Neonatal sepsis is historically categorised as either early-onset sepsis (EOS) or late-onset sepsis (LOS), with EOS variably defined as sepsis within 72 hours or up to 7 days of birth. EOS is traditionally thought to be caused by organisms such as group B streptococcus and enteric Gram-negative bacteria, acquired peripartum from the maternal genital tract. LOS, on the other hand, is considered to arise due to the acquisition of pathogens during hospitalisation, with very low birth weight and early gestational age being strong risk factors [4]. Increasingly, this distinction is called into question by reports, particularly from low- and middle-income countries (LMICs), of a predominance (up to 64% [5]) of Gram-negative and hospital-associated infections in both EOS and LOS [4–6]. There has been a worldwide increase in the prevalence of Gram-negative neonatal sepsis, with an alarming upward trend in multidrug-resistant (MDR) infections [4–7]. It has been estimated that globally 214,000 neonatal sepsis deaths are attributable to resistant pathogens each year [8]. Access to antimicrobials remains a significant barrier for many neonates and children in LMICs [8] and has resulted in an increase in neonatal mortality [9].

The World Health Organization (WHO) recommends the use of gentamicin with either ampicillin or benzylpenicillin as first-line treatment for neonatal and paediatric sepsis in resource-limited settings, with ceftriaxone as recommended second-line therapy [10]. Recent systematic reviews of antimicrobial resistance (AMR) in neonates and children in sub-Saharan Africa have highlighted the increasing prevalence of resistance to these antibiotics, particularly in Gram-negative bacteria [11,12]. As a consequence, antibiotic prescribing practices in neonatal and paediatric sepsis have been shown to significantly diverge from the WHO recommendations [13]. In light of the increasing evidence of multidrug resistance in Gram-negative neonatal sepsis, we performed a systematic review of published data on Gram-negative neonatal sepsis from all low- and lower-middle-income countries (LLMICs) from 1 January 2010 to 21 April 2021 to assess the appropriateness of current WHO first- and second-line antimicrobial recommendations.

## Methods

### Search strategy and selection criteria

This systematic literature search and review was performed using a predesigned study protocol (published in PROSPERO, CRD42020181110) and adheres to the PRISMA guideline for reporting systematic reviews and meta-analyses (see S1 Table). We searched Ovid MEDLINE, Embase, PubMed, CENTRAL (Cochrane Central Register of Controlled Trials), Web of Science, LILACS (Latin American and Caribbean Health Sciences Literature), WHOLIS, MedCarib, African Journals Online, African Index Medicus, IMSEAR (Index Medicus for South-East Asia Region), IMEMR (Index Medicus for the Eastern Mediterranean Region), WPRIM (Western Pacific Region Index Medicus), IndMED, Google, and OpenGrey to identify studies published from 1 January 2010 to 21 April 2021 (date of last search) that reported aetiology of neonatal sepsis (bacteraemia, sepsis, septicaemia, or meningitis) in LLMICs. Bibliographies of published systematic reviews were also assessed for eligibility (snowball method). Included studies needed to specifically address neonatal data or provide neonatal data that were clearly distinguishable from other age groups. To be inclusive, we defined neonates as infants up to 3 months of age, and neonatal sepsis was defined as neonates with clinical signs and/or laboratory evidence of sepsis. Countries were defined as low income and lower middle income according to the World Bank in June 2019 [14], and this categorisation was applied across the entire review period. Search terms were developed in accordance with PICOS (population, intervention, control, outcome, and setting) domains, and each database was searched using various combinations of the following terms: ‘infant, newborn’, ‘sepsis’, ‘meningitis’, ‘Gram-negative aerobic bacteria’, ‘Gram-negative bacteria’, ‘Gram-negative bacterial infections’, ‘microbial sensitivity tests’, ‘drug resistance’, and ‘developing countries’. These terms were applied to the title and abstract of publications. LLMICs per the World Bank 2019 list were also searched individually in Google with.org and.gov domains to include relevant materials from local shelves. The full search strategy and details of the quality assessment performed on each article can be found in S1 Text and S2 Table.

Studies were excluded if they presented aggregated data from which country-specific data could not be clearly identified, or from which neonates could not clearly be distinguished from older children or adults. Studies were also excluded if they presented insufficient information on the type of Gram-negative organisms or antimicrobial susceptibility, or if they reported infections of non-sterile sites. To ensure data were relevant to the current epidemiology of neonatal sepsis in LLMICs, a decision was made to exclude studies prior to 2010 and studies reporting a majority of isolates prior to 2010. Case reports or series (studies of fewer than 10 patients or with fewer than 10 Gram-negative isolates in total) were excluded due to study design, as they were likely to have significant biased selection of participants. Abstracts and titles were compiled in Endnote, and duplicates were removed. Two investigators (SCHW and YE) individually reviewed the identified articles to determine eligibility. All eligible articles were retrieved in full text. For references where we were unable to retrieve the full text and those with results that required clarification to assess eligibility, direct email contacts were sent to the corresponding authors. Non-English articles were included if data were able to be reliably extracted using Google translate. Disagreement over inclusion was resolved by consensus.

### Data extraction

A data extraction checklist was developed based on the PICOS domains. Population variables included demographics of the neonatal population (sex, gestational age, and median age) and definition of neonatal sepsis (clinical or laboratory based). Outcome variables included timing of neonatal sepsis (early or late, and proportion with positive cultures for each category), total number of neonatal sepsis cases, mortality rate, number of blood or cerebrospinal fluid (CSF) cultures performed and number of positive cultures, microbiological methods, number of Gram-negative organisms identified (specifically, number of *E*. *coli*, *Klebsiella* species, *Enterobacter* species, *Pseudomonas* species, and *Acinetobacter* species), results of antimicrobial susceptibility testing (ampicillin, gentamicin, amikacin, third-generation cephalosporins [3GC], ciprofloxacin, and carbapenem), and burden of extended-spectrum beta-lactamases. Setting variables included country, city, setting (community, hospital-based [including neonatal intensive care], special care baby unit, or paediatric ward), study design, publication year, and study years. Two investigators (SCHW and LR) independently extracted the above variables into an Excel spreadsheet.

### Quality assessment

The quality of each article was assessed independently by 2 investigators (SCHW and LR) using either the Newcastle–Ottawa quality assessment tool (case–control, cross-sectional, and cohort studies) or Cochrane risk of bias tool randomised controlled trials. Consensus was reached by panel discussion between 3 investigators (SCHW, LR, and YE). The results of quality assessment are summarised in S2 Table.

### Statistical analysis

Meta-analysis was conducted to calculate pooled prevalence of positive blood or CSF culture, and of the 5 major Gram-negative bacterial species using the ‘metaprop’ command of the ‘metan’ package in Stata 16 [123]. Pooled prevalence of resistance against 6 key antimicrobials for each major Gram-negative bacterial species was also calculated with this method. Ampicillin resistance was not reported for *Klebsiella*, *Enterobacter*, *Pseudomonas*, and *Acinetobacter* spp. as these bacteria are intrinsically resistant. Stratification was done by continent of study (Asia versus Africa). Pooled prevalence was calculated as effect size with 95% confidence intervals (CIs) using logistic-normal random-effect models. Given the variability of the patient characteristics within the studies, the random-effect model was applied irrespective of the *I*^2^ statistics.

### Publication bias and study heterogeneity

Sensitivity and subgroup analyses as well as meta-regression models were used to investigate sources of heterogeneity and the factors that affect the magnitudes of estimates, where data were available. The sensitivity analyses were conducted by excluding 1 study each time and recalculating the pooled prevalence. Funnel plots and Egger’s meta-regression test were used to assess small study effects. Study heterogeneity was reported using the *I*^2^ measure of inconsistency.

All statistical analyses were done using Stata 16 (StataCorp, 2015). The results are illustrated on a world map, using data from the public domain map dataset Natural Earth (https://www.naturalearthdata.com), through the ‘rnaturalearth’ package (version 0.1.0) [16] in R 3.6.0 [17].

## Results

**Fig 1:**
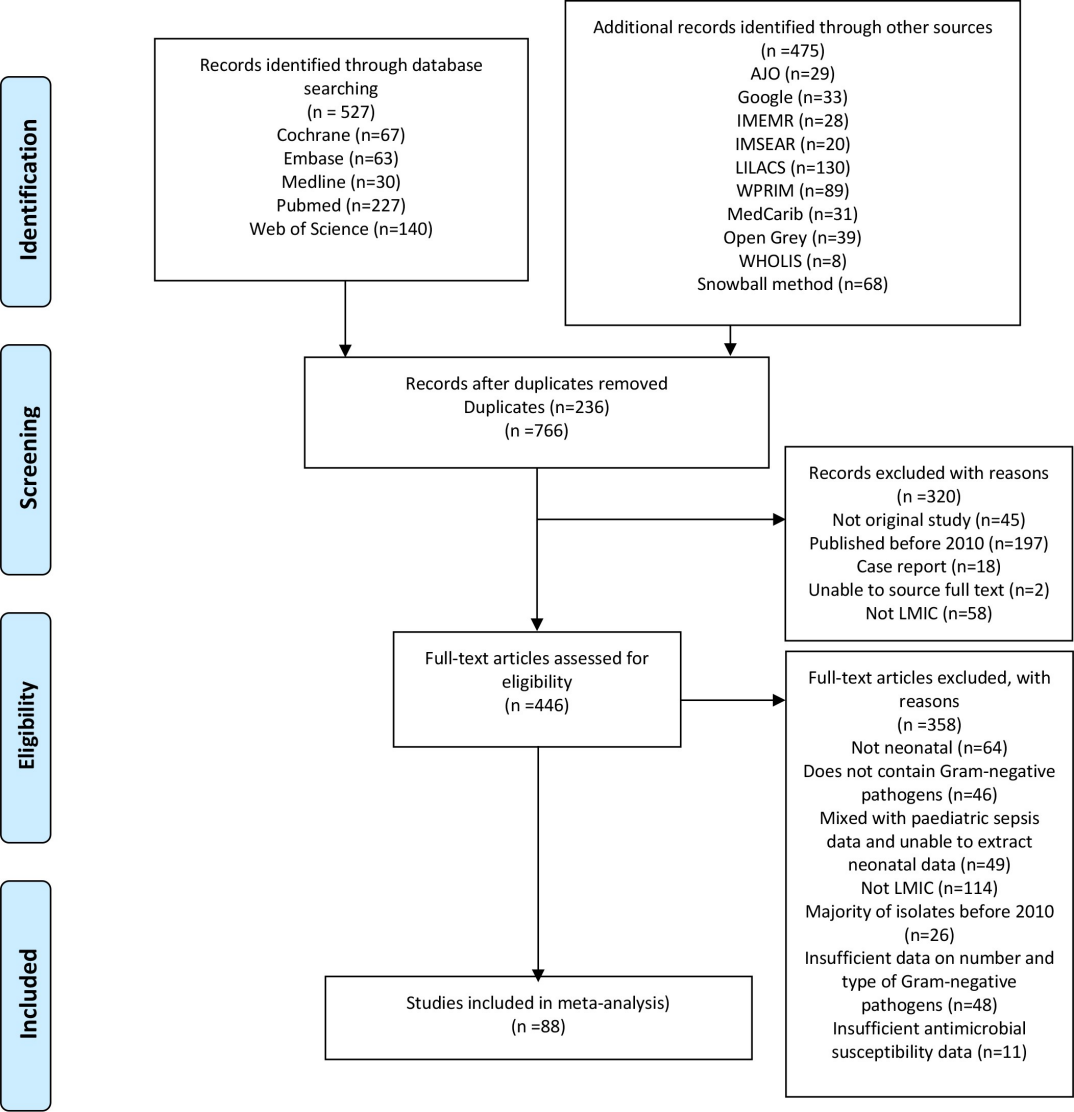
Study selection. AJO, African Journals Online; IMEMR, Index Medicus for the Eastern Mediterranean Region; IMSEAR, Index Medicus for South-East Asia Region; LILACS, Latin American and Caribbean Health Sciences Literature; LMIC, low- or middle-income country; WPRIM, Western Pacific Region Index Medicus.

**Fig 2:**
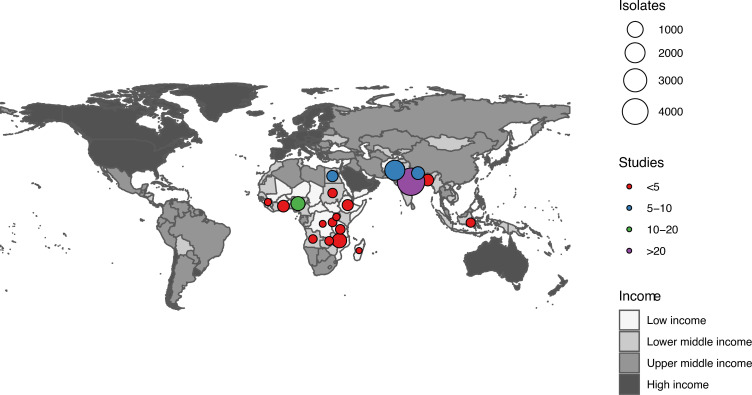
Distribution of included studies in low- and lower-middle-income countries. The world map was created using data from the public domain map dataset Natural Earth (https://www.naturalearthdata.com).

Our search yielded 766 results, of which 446 studies were eligible for full-text screening; 358 studies were excluded after full-text screening, and 88 studies were included in the full synthesis (Fig 1). Two studies were published in Bahasa Indonesian and 1 in French, with the remainder in English. There was no evidence of asymmetry by Egger’s meta-regression test (S1 Data). There were 34 studies from the Africa region—Angola (1) [18], Congo (1) [19], Egypt (5) [20–24], Ethiopia (4) [25–28], Ghana (1) [29], Guinea (1) [30], Madagascar (1) [31], Malawi (1) [32], Nigeria (12) [28,33–43], Rwanda (1) [28], Sudan (1) [15], Tanzania (3) [44–46], Uganda (1) [47], and Zambia (1) [48]—and 59 studies from the Asia region—Bangladesh (4) [28,49–51], India (37) [5,28,52–86], Indonesia (2) [87,88], Nepal (9) [89–97], and Pakistan (7) [28,98–103]—as depicted in Fig 2. One publication included data from 6 countries [28]. There were no studies identified from LLMICs outside the Africa or Asia regions. Most studies (*n =* 81) used a cross-sectional design. Study settings were reported in 84 studies, with a majority of the studies undertaken in a hospital setting. Forty-nine studies reported data from neonatal intensive care units (NICUs), 10 studies from special care baby units, 22 studies from a paediatric ward or unspecified hospital setting, and only 3 studies from the community. The definition of EOS was documented in 58 studies (66%): sepsis occurring within 48 hours of birth (1 study), within 72 hours of birth (43 studies), and within 1 week of birth (14 studies). There was also variation in the definition of the neonatal period (reported in 45 studies): 0–4 days (1 study), 0–28 days (38 studies), 0–30 days (2 study), and 0–60 days (4 studies). Bacteriological identification and antimicrobial susceptibility testing methods were reported in 70 studies (80%) and 76 studies (86%), respectively. Disk diffusion was the most commonly reported antimicrobial susceptibility testing method (70/72 studies). Most studies included data on all major Gram-negative species. Five studies reported on a subgroup of Gram-negative bacteria (3 studies on *Acinetobacter* spp. [*n =* 223] and 2 studies on Enterobacterales [*n =* 273]). Significant heterogeneity was observed across all meta-analyses and subgroup analyses. We could not identify explicit sources of heterogeneity due to the limitations of the available data. The characteristics of all included studies are summarised in S3 Table.

**Fig 3:**
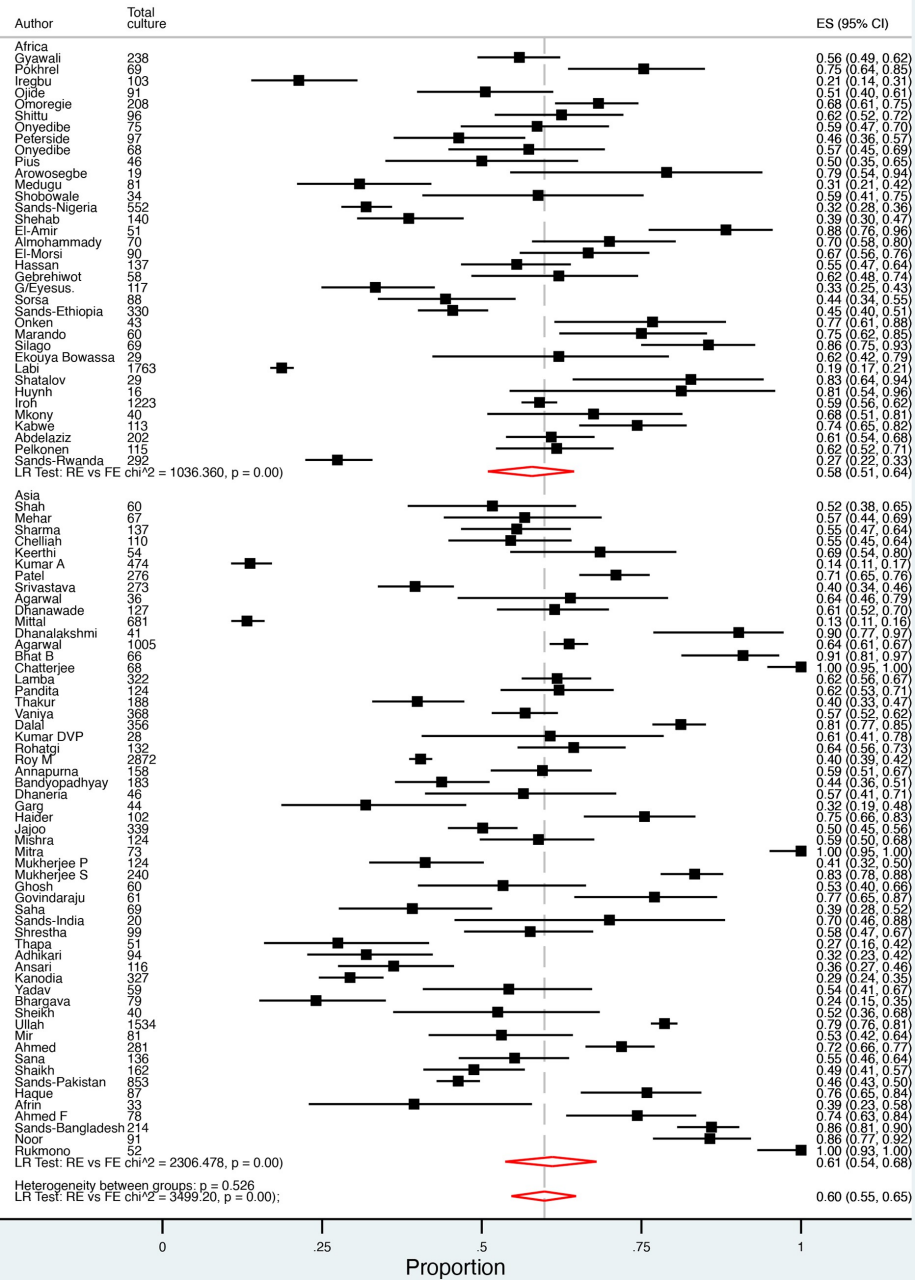
Pooled prevalence of Gram-negative neonatal sepsis. ES, effect size; FE, fixed effect; LR, likelihood ratio; RE, random effect.

The included studies of neonatal sepsis in LLMICs documented 20,828 positive blood/CSF cultures. The culture positivity prevalence ranged from 3% to 88% across 82 studies (denominator data missing for 6 studies). In 43 studies, a median of 60% (range 26% to 95%) of positive blood/CSF cultures were reported to have been taken in the study’s defined period of EOS. In 21 studies, a median of 62% (range 13% to 82%) of positive blood/CSF cultures were from premature neonates. The estimated pooled proportion of neonatal sepsis caused by Gram-negative organisms was 60% (95% CI 55% to 65%, *I*^2^ 97%), and was 58% (95% CI 51% to 64%, *I*^2^ 97%) and 61% (95% CI 53% to 66%, *I*^2^ 98%) for Africa and Asia, respectively (Fig 3). *Klebsiella* spp. accounted for 38% (95% CI 33% to 43%, *I*^2^ 96%) of Gram-negative neonatal sepsis, followed by 15% *E*. *coli* (95% CI 12% to 18%, *I*^2^ 95%), 7% *Pseudomonas* spp. (95% CI 5% to 9%, *I*^2^ 89%), 6% *Acinetobacter* (95% CI 4% to 10%, *I*^2^ 96%), and 3% *Enterobacter* spp. (95% CI 2% to 5%, *I*^2^ 86%). We observed a higher proportion of neonatal sepsis caused by *Klebsiella* spp. in Africa than Asia (44% versus 35%, with *I*^2^ 90% and 96%, respectively), while *Acinetobacter* was more commonly reported in Asia than Africa (10% versus 3%, *I*^2^ 98% and 79%, respectively). Significant heterogeneity was noted with these findings (S2 Data).

**Fig 4:**
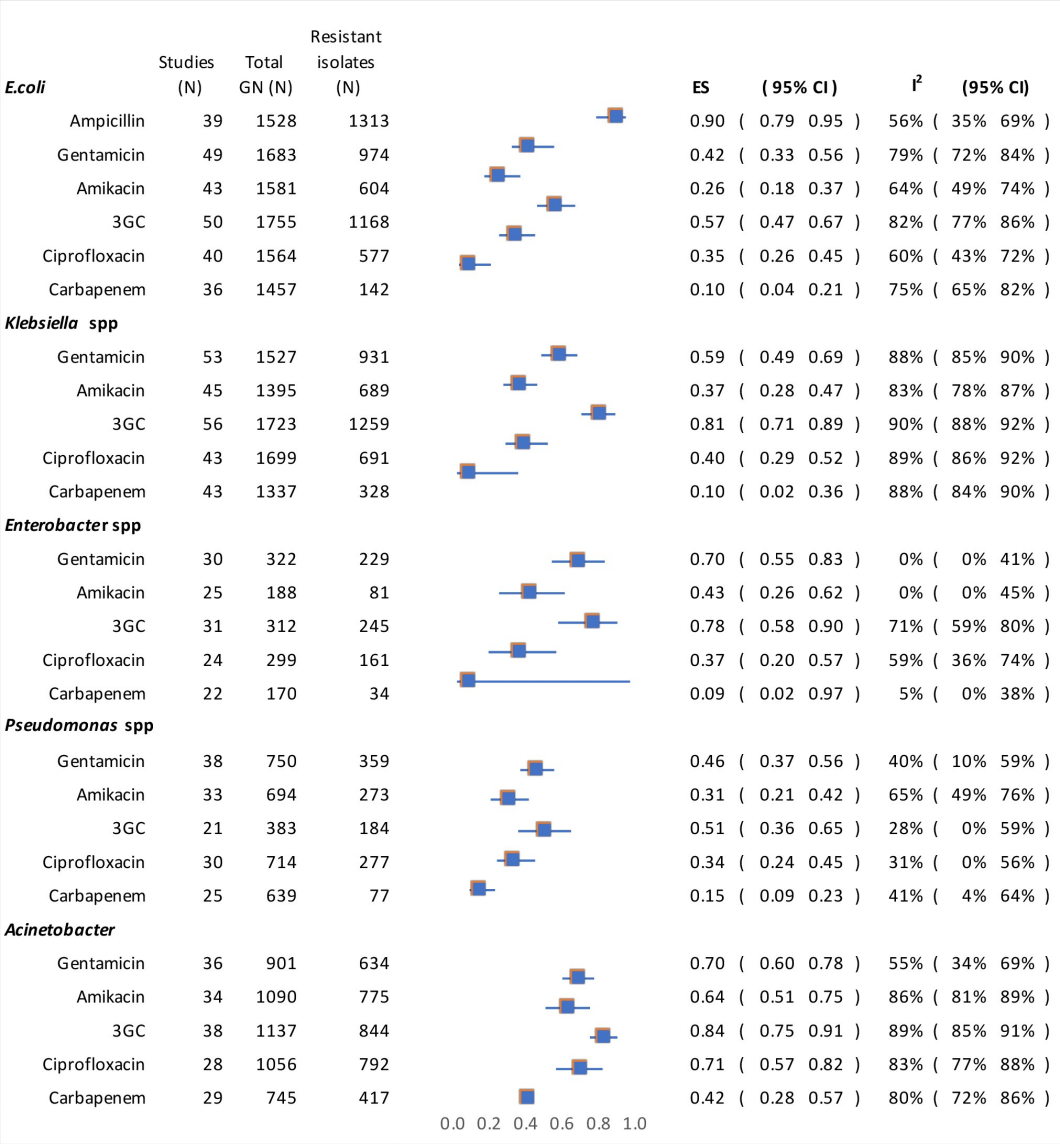
Pooled prevalence of antimicrobial resistance. 3GC, third-generation cephalosporin; ES, effect size; GN, Gram-negative.

The pooled prevalence estimates of resistance to ampicillin, gentamicin, amikacin, 3GC, ciprofloxacin, and carbapenem are shown in Fig 4. Substantial resistance to gentamicin (from 42% to 70%) was observed in each of the specified Gram-negative species. Similarly, high levels of resistance to ceftriaxone were noted in *E*. *coli*, *Klebsiella* spp., *Enterobacter* spp., and *Acinetobacter* spp. (57% to 81%). We observed a higher prevalence of 3GC resistance in Africa compared to Asia, particularly with *Klebsiella* and *Pseudomonas* spp.; however, there was significant heterogeneity for these findings. Pooled prevalence of 3GC resistance in Africa for *Klebsiella* spp. was 88% (95% CI 72% to 96%, *I*^2^ 92%) versus 77% in Asia (95% CI 65% to 87%, *I*^2^ 90%). For *Pseudomonas* spp., 3GC resistance was 59% (95% CI 34% to 80%, *I*^2^ 9%) in Africa versus 46% in Asia (95% CI 28% to 65%, *I*^2^ 45%). The prevalence of ciprofloxacin resistance was higher in Asia across all 5 key groups of Gram-negative bacteria compared to Africa (37% to 76% versus 20% to 44%; S4 Table). The overall prevalence of extended-spectrum beta-lactamases was reported in 10 studies and ranged widely, from 14% to 95%. The overall pooled estimate of carbapenem resistance was 10% for *E*. *coli* (95% CI 4% to 21%, *I*^2^ 75%), 10% for *Klebsiella* spp. (95% CI 2% to 36%, *I*^2^ 88%), 15% for *Pseudomonas* spp. (95% CI 9% to 23%, *I*^2^ 41%), and 42% for *Acinetobacter* spp. (95% CI 28% to 57%, *I*^2^ 80%; S4 Table).

## Discussion

Our systematic review and meta-analysis identified that in LLMICs, approximately 60% of cases of neonatal sepsis were caused by Gram-negative bacteria. The prevalence of resistance to the WHO-recommended first-line antimicrobials ampicillin and gentamicin in 5 common groups of Gram-negative neonatal sepsis organisms is over 90% and 40%, respectively. Resistance to 3GC is also highly prevalent and of substantial concern.

This review has several limitations. To facilitate meaningful data interpretation about the current epidemiology of neonatal sepsis in LLMICs, a decision was made to exclude studies prior to 2010. There may be potential sampling bias as most of the studies in this review are from tertiary-level urban hospitals, which could lead to an overestimate of the burden caused by Gram-negative bacteria. Studies of AMR in children [12,104,105] have highlighted the lack of differentiation between community- and hospital-acquired infections as an issue, which we have similarly found. For most of the studies included, the sources of positive blood cultures were not reported, and therefore we were unable to determine the relative proportions of community- and hospital-acquired infections. This could be significant as there may be important epidemiological and AMR profile differences, depending on setting. Efforts to improve maternal and neonatal mortality in LMICs by increasing the numbers of infants delivered in healthcare facilities may increase exposure to antimicrobial-resistant organisms [106]. The focus of this study is to provide a snapshot on AMR in the least resourced countries. We have limited our search to LLMICs and acknowledge that this fails to address the issue of AMR in other middle-income countries. We applied the World Bank 2019 income categories across the entire review period and may have inadvertently missed studies reporting data from LLMICs that subsequently transitioned to a higher income category. Our findings may have less relevance for the empirical approach to ‘possible serious bacterial infection’ as defined by the WHO. This much broader group of acute infections, in which pathogens are rarely cultured even when referral to hospital is achieved, exhibit much lower mortality using narrow-spectrum penicillins and gentamicin [107,108]. Despite the focus on tertiary-level facilities, previous studies have noted a variable quality of microbiological data from studies in LMICs [109]. In this review, we report the microbiological methods used where these are documented, alongside the reported methods used to measure antimicrobial susceptibility, largely disc diffusion methods, as per the Clinical and Laboratory Standards Institute. The gold standard of laboratory accreditation by the International Organization for Standardisation may be challenging to achieve for laboratories in resource-limited facilities in LMICs [110]. Our findings further stress the importance of quality improvement initiatives such as Strengthening Laboratory Management Toward Accreditation and WHO’s Laboratory Quality Stepwise Implementation tool.

There was significant heterogeneity among the included studies, reflecting the differences in geography, case ascertainment, and microbiological and data collection methods. Some pooled estimates of AMR were derived from studies with small numbers of isolates and highly variable blood culture positivity rates, resulting in residual uncertainty about the precision of these estimates. These limitations are important when interpreting our findings. Whilst we did not detect any significant effect from small studies using a conventional funnel plot, there was clear evidence of a geographical publication bias. There are 78 countries categorised as LLMICs by the World Bank, and we identified data from only 19 countries (all within Asia and Africa). There was a predominance of studies from South Asia in the Asian continent, and care must be taken not to extrapolate these results to other countries in the region where there were no data available. Significant regional gaps were also noted from the Americas and the Western Pacific region, with no studies available for inclusion from the Pacific Island countries and territories. It has been estimated that a newborn infant dies every 2 minutes in the Western Pacific region, with infection being an important cause of death [111]. Yet there are no published data available from many countries in this region to help us understand the infectious aetiology of neonatal sepsis. Recent reviews from Pacific Island countries and territories confirm the presence of MDR organisms in the region [112], but the capacity for structured AMR surveillance and reporting is limited, which leads to challenges in interpretation of the findings [113]. It has been previously noted that systematic reviews aiming to include evidence from LMICs face challenges in accessing non-English literature and require searches of less well-known regional databases, particularly for grey literature [114]. We made extensive efforts to identify eligible studies in regional databases. We identified a single potentially eligible study from the Americas but were unable to obtain data on request from the corresponding author.

Our findings echo similar reports from systematic reviews focussing on sub-Saharan Africa and South Asia [11,12,115]. *Klebsiella* spp. was the most common causative Gram-negative bacteria, accounting for 38% of Gram-negative neonatal sepsis. This predominance appears more pronounced in studies from LMICs in Africa, which is also consistent with previous reports [11,12]. Our finding of a higher prevalence of *Acinetobacter* spp. in Asia is both interesting and of substantial concern. This observation may reflect the NICU setting of these studies (over 60% of studies in Asia were from NICUs), with increased likelihood of early invasive interventions, particularly in premature infants. Similarly, there is a high rate of cesarean delivery reported in South Asia [116], which may impact on maternal–neonatal acquisition of MDR *Acinetobacter* during hospitalisation. Of note, the Aetiology of Neonatal Infections in South Asia (ANISA) study [107], one of the very few community-based studies of neonatal sepsis, also reported Gram-negative organisms as the predominant cause of serious neonatal bacterial infections. Similar studies in other LMICs will help to assess whether there are any significant differences between hospital- and community-acquired neonatal sepsis.

Inappropriate empirical antibiotic therapy has recently been shown to be associated with increased mortality in young children and neonates, highlighting the importance of appropriate empirical antibiotic recommendations [117]. WHO-recommended first-line antibiotics for neonatal and paediatric sepsis are ampicillin and gentamicin, with ceftriaxone being second-line. Our finding of a high level of resistance against gentamicin across all key groups of Gram-negative bacteria raises the question of the appropriateness of its inclusion in empirical neonatal sepsis treatment regimens for LMICs, particularly in the hospital-based setting. Rates of resistance to ceftriaxone are similarly concerning. The most recent report from the BARNARDS observational cohort study of neonatal sepsis and AMR in 6 LMICs also found that only 28.5% of Gram-negative isolates were susceptible to at least 1 antibiotic in the combination of ampicillin and gentamicin, and declared that ampicillin is now redundant for treating neonatal sepsis in LMICs, with 97% of Gram-negative isolates resistant to ampicillin [118]. The prescribing practices of clinicians may reflect these findings of high levels of gentamicin and ceftriaxone resistance. A recent global point prevalence survey of antimicrobial prescribing in neonatal and paediatric sepsis identified that less than a quarter of neonates received WHO-recommended first- or second-line empirical antibiotics for sepsis [13]. In LMICs, meropenem was the most common empirical antibiotic prescribed for sepsis in hospitalised neonates and children (15.9% of antibiotic regimens prescribed) [13]. This may be appropriate given the local epidemiology, as suggested by the findings in this systematic review and meta-analysis. Region- or country-specific empirical antibiotic regimens for neonatal sepsis are indicated, which further highlights the need for structured AMR surveillance and reporting in LMICs, as these data are required to inform the most appropriate local recommendations. It is worthwhile noting that in some LMICs, AMR surveillance and reporting are impossible due to the lack of access to blood cultures [119]. Rapid diagnostics for infections are not novel to LMICs, with point-of-care testing readily available for conditions such as HIV and malaria. Rapid diagnostics including culture-independent methods for bloodstream infections and AMR may have an important role in LMICs. The development of low-cost tests that do not require significant laboratory infrastructure should be prioritised [120]. Tests that facilitate timely identification of causative pathogens and antibiotic resistance mechanisms may guide empirical antibiotic choice for neonatal sepsis, and improve antimicrobial stewardship by reducing empirical broad-spectrum antibiotic use.

Our findings provide important insight into the role of Gram-negative pathogens in neonatal sepsis in LLMICs, the burden of AMR in this context, and the appropriateness of existing recommendations for antimicrobial therapy. With limited access to third-line therapies (such as carbapenems) and the development of resistance even to these, robust surveillance of infection and AMR, along with infection prevention and antimicrobial stewardship strategies, is critical to address this global health threat [9]. An increase in the rate of facility births and neonatal interventions across many LMICs further highlights the importance of infection control and prevention. Dedicated cleaning interventions can improve cleanliness and reduce the burden of contaminated surfaces in low-resource NICUs [121]. The types of Gram-negative pathogens and associated AMR patterns are likely to evolve over time, and consideration should be given to the development of platforms that provide these data in a useful format and timely fashion. This will facilitate dynamic review of existing recommendations and their appropriateness. New antimicrobial strategies against MDR Gram-negative organisms appropriate for LLMICs need to be prioritised in parallel. The optimal dosing and duration of treatment with repurposed and new antimicrobials effective against MDR Gram-negative bacterial infections are frequently unknown for neonates [122]. There is a clear ethical mandate to prioritise trials of antimicrobials in neonates born in countries with the highest burden of AMR, and to ensure that antimicrobials are made accessible to clinicians and families in LLMICs.

### Conclusion

Neonatal sepsis is increasingly caused by Gram-negative bacteria with alarming rates of multidrug resistance. Mortality is increased in neonatal sepsis caused by MDR organisms. The development of robust AMR surveillance and reporting in LLMICs should be prioritised to underpin region-specific empirical antimicrobial recommendations. There is an urgent need for high-quality antimicrobial trials in neonates and ensuring equitable access to new and effective antimicrobials.

## Supporting information

S1 Data Publication bias assessment. (DOCX)

S2 Data Prevalence of key Gram-negative bacteria species. (DOCX)

S1 Table PRISMA checklist. (DOCX)

S2 Table Risk of bias assessment (individual studies). (DOCX)

S3 Table Study characteristics. (DOCX)

S4 Table Pooled antimicrobial resistance rates. (DOCX)

S1 Text Search strategy. (DOCX)

## Abbreviations

3GC: third-generation cephalosporin
AMR: antimicrobial resistance
CSF: cerebrospinal fluid
EOS: early-onset sepsis
LLMICs: low- and lower-middle-income countries
LMICs: low- and middle-income countries
LOS: late-onset sepsis
MDR: multidrug-resistant
NICU: neonatal intensive care unit
WHO: World Health Organization
