# Supplementary material for: Gram-negative neonatal sepsis in low- and lower-middle-income countries and WHO empirical antibiotic recommendations: A systematic review and meta-analysis
Source: PLoS Med. 2021 Sep 28;18(9):e1003787. doi: 10.1371/journal.pmed.1003787 (PMC8478175; doi:10.1371/journal.pmed.1003787)
Supplement: S1 Data — (DOCX) [file pmed.1003787.s001.docx]

| Results from the Egger’s meta-regression test assessing the presence of small-study effect in the # studies investigation Neonatal Sepsis caused by Gram-negative bacteria | | | | | | |
| --- | --- | --- | --- | --- | --- | --- |
| Parameter | Estimate | SE | T Value | P Value | 95% CI | |
| Slope (coefficient) | -.3218 | 0.5034 | 6.39 | 0.00 | 0.2217 | 0.42185 |
| Bias (intercept) | 7.7099 | 3.5410 | 2.18 | 0.032 | 0.6729 | 14.7470 |
| Test of Ho: no small-study effects, p value=0.000 | | | | | | |

We appreciate that degree of prevalence spread is not a direct reflection of publication bias. We conducted the Egger’s meta-regression model to investigate the magnitude and statistical significance of effect of small studies. The test result shows p-value less than 0.05 and 95% CI doesn’t across 1, suggesting that there is no evidence of publication bias.
