# Supplementary material for: Gram-negative neonatal sepsis in low- and lower-middle-income countries and WHO empirical antibiotic recommendations: A systematic review and meta-analysis
Source: PLoS Med. 2021 Sep 28;18(9):e1003787. doi: 10.1371/journal.pmed.1003787 (PMC8478175; doi:10.1371/journal.pmed.1003787)
Supplement: S2 Table — (DOCX) [file pmed.1003787.s004.docx]

RCT
