# Supplementary material for: Gram-negative neonatal sepsis in low- and lower-middle-income countries and WHO empirical antibiotic recommendations: A systematic review and meta-analysis
Source: PLoS Med. 2021 Sep 28;18(9):e1003787. doi: 10.1371/journal.pmed.1003787 (PMC8478175; doi:10.1371/journal.pmed.1003787)
Supplement: S3 Table — (DOCX) [file pmed.1003787.s005.docx]

| **Author/Year of publication** | **Country** | **Study setting** | **Study design** | **Year(s) of isolates** | **Definition of EOS** | **Age of neonates included** | **EOS in positive cultures**  **(n/N, %)** | **Positive cultures with premature neonates** | **Bacteriologic identification method** | **Antimicrobial susceptibility method** |
| --- | --- | --- | --- | --- | --- | --- | --- | --- | --- | --- |
| **Asia** | | | | | | | | | | |
| **Adhikari 2014** | Nepal | Multiple inpatient settings | Cross sectional | 2011-2012 | EOS <72hrs | NR | 89/94 (95%) | 63/94 (67%) | Phenotypic & biochemical | Disk diffusion |
| **Afrin 2016** | Bangladesh | NICU | Cross sectional | 2014-2015 | Not defined | 0-28 days | NR | NR | Phenotypic & biochemical | Disk diffusion |
| **Agarwal 2015** | India | NICU | Cross sectional | 2014 | EOS <72hrs | NR | 22 /36 (61%) | 23/36 (64%) | Phenotypic & biochemical | Disk diffusion & Vitek |
| **Agarwal 2016** | India | NICU | Cohort study | 2011-2014 | EOS <72hrs | NR | 1351/1980 (68%) | NR | NR | NR |
| **Ahmed 2018** | Pakistan | NICU | Cross sectional | 2017 | Not defined | NR | 182/281 (65%) | NR | NR | NR |
| **Ahmed F 2018** | Bangladesh | NICU | Cross sectional | 2012-2016 | Not defined | NR | NR | NR | NR | NR |
| **Annapurna 2018** | India | NICU | Cross sectional | 2016 | Not defined | 0-28 days | NR | NR | NR | NR |
| **Ansari 2015** | Nepal | Paediatric ward | Cross sectional | 2012-2013 | EOS <72hrs | 0-28 days | 82/116 (71%) | NR | Phenotypic & biochemical | Disk diffusion |
| **Bandyopadhyay 2018** | India | NICU | Cross sectional | 2012-2014 | Not defined | NR | 108/183 (59%) | 141/183 (77%) | NR | NR |
| **Bhargava 2020** | Nepal | NICU | Cross sectional | 2019 | EOS <72hrs | 0-28 days | 60/79 (76%) | NR | Phenotypic & biochemical | Disk diffusion |
| **Bhat B 2016** | India | NICU | RCT | 2013 | Not defined | NR | NR | NR | NR | NR |
| **Chatterjee 2016** | India | NICU | Cross sectional | 2007-2014 | Not defined | NR | 39/63 (62%) | 56/68 (82%) | Phenotypic & biochemical | Disk diffusion & Vitek |
| **Chelliah 2014** | India | Not stated | Cross sectional | 2010-2011 | EOS <72hrs | NR | 86/110 (78%) | 14/110 (13%) | Phenotypic & biochemical | Disk diffusion |
| **Dalal 2017** | India | NICU | Cross sectional | 2010-2013 | EOS <72hrs | NR | 178/356 (50%) | NR | NR | Disk diffusion |
| **Dhanalakshmi 2015** | India | SCBU/premature baby unit | Cross sectional | 2013-2014 | Not defined | NR | NR | NR | Phenotypic & biochemical | Disk diffusion |
| **Dhanawade 2015** | India | NICU | Cross sectional | 2012-2014 | EOS <72hrs | NR | 65/127 (51.2%) | 96/127 (76%) | Phenotypic & biochemical | Disk diffusion |
| **Dhaneria 2018** | India | NICU | Cross sectional | 2012-2014 | EOS <72hrs | NR | NR | NR | Phenotypic & biochemical | Disk diffusion |
| **Garg 2018** | India | Multiple inpatient settings | Cross sectional | 2016 | Not defined | 0-28 days | 41/44 (93.2%) | 21/44 (48%) | Phenotypic & biochemical | Disk diffusion |
| **Ghosh 2020** | India | SCBU/premature baby unit | Cross sectional | 2017-2018 | EOS <1wk | 0-28 days | 21/60 (35%) | NR | Phenotypic & biochemical | Disk diffusion |
| **Govindaraju 2020** | India | NICU | Cross sectional | 2015-2018 | EOS <72hrs | 0-28 days | 16/61 (26%) | NR | NR | NR |
| **Gyawali 2013** | Nepal | Multiple inpatient settings | Cross sectional | 2009-2010 | Not defined | 0-28 days | NR | NR | Phenotypic & biochemical | Disk diffusion |
| **Haider 2018** | India | NICU | Cross sectional | 2017-2018 | Not defined | NR | 75/102 (74%) | 63/102 (62%) | Phenotypic & biochemical | Disk diffusion & broth microdilution |
| **Haque 2014** | Bangladesh | NICU | Cross sectional | 2011 | EOS <1wk | 0-28 days | 66/87 (76%) | 46/87 (53%) | Phenotypic & biochemical | Disk diffusion |
| **Hassan2020** | Egypt | NICU | Cross sectional | 2017-2017 | Not defined | 0-28 days | NR | NR | Phenotypic & biochemical | Disk diffusion |
| **Jajoo 2018** | India | Community | Cohort study | 2011-2015 | EOS <72hrs | NR | NR | NR | Phenotypic & biochemical | Disk diffusion & broth microdilution |
| **Kanodia 2017** | Nepal | Paediatric ward | Cross sectional | 2014 | Not defined | NR | NR | NR | Phenotypic & biochemical | Disk diffusion |
| **Keerthi 2014** | India | NICU | Cross sectional | 2010 | EOS <1wk | NR | 42/54 (78%) | NR | NR | NR |
| **Kumar A 2014** | India | Paediatric ward | Cross sectional | 2010-2012 | Not defined | NR | NR | NR | Phenotypic & biochemical | VITEK |
| **Kumar DVP 2017** | India | SCBU/premature baby unit | Cross sectional | 2013-2015 | EOS <72hrs | 0-28 days | NR | NR | Phenotypic & biochemical | Disk diffusion |
| **Lamba 2016** | India | Multiple inpatient settings | Cross sectional | 2014-2015 | EOS <72hrs | 0-28 days | NR | NR | Phenotypic & biochemical | Disk diffusion |
| **Mehar 2013** | India | NICU | Cross sectional | 2012 | EOS <72hrs | NR | 36/63 (57%) | 39/67 (58%) | Vitek | Vitek |
| **Mir 2017** | Pakistan | Community | RCT | 2010-2013 | EOS <1wk | 0-60 days | NR | NR | Phenotypic & biochemical | Disk diffusion |
| **Mishra 2018** | India | Multiple inpatient settings | Cross sectional | 2015-2017 | EOS <72hrs | 0-28 days | 59/124 (47.6%) | NR | Phenotypic & biochemical | Disk diffusion |
| **Mitra 2019** | India | NICU | Cross sectional | 2012-2014 | EOS <72hrs | 0-28 days | NR | NR | Phenotypic & biochemical | Disk diffusion & E-test |
| **Mittal 2015** | India | NICU | Cross sectional | 2012 | Not defined | NR | NR | NR | Phenotypic & biochemical | Disk diffusion & Vitek |
| **Mukherjee P 2019** | India | Multiple inpatient settings | Cross sectional | 2016-2017 | EOS <72hrs | 0-28 days | 33/124 (27%) | NR | Phenotypic & biochemical | Disk diffusion |
| **Mukherjee S 2019** | India | Multiple inpatient settings | Cross sectional | 2016-2017 | Not defined | NR | NR | NR | Phenotypic & biochemical | Disk diffusion |
| **Noor 2012** | Indonesia | NICU | Cross sectional | 2010-2011 | EOS <72hrs | NR | NR | NR | Phenotypic & biochemical | Disk diffusion |
| **Pandita 2016** | India | NICU | Cross sectional | 2013-2015 | EOS <72hrs | 0-28 days | NR | NR | NR | Disk diffusion |
| **Patel 2014** | India | NICU | Cross sectional | 2007-2011 | Not defined | NR | NR | NR | API | Vitek |
| **Pokhrel 2018** | Nepal | NICU | Cross sectional | 2014-2017 | EOS <72hrs | NR | 54/69 (78%) | 47/69 (68%) | Phenotypic & biochemical | Disk diffusion |
| **Rohatgi 2017** | India | NICU | RCT | 2012-2014 | Not defined | NR | 75/132 (57%) | 72/132 (55%) | Phenotypic & biochemical | Disk diffusion |
| **Roy M 2017** | India | NICU | Cross sectional | 2009-2014 | Not defined | NR | NR | NR | NR | Disk diffusion |
| **Rukmono 2016** | Indonesia | NICU | Cross sectional | 2010 | Not defined | NR | NR | NR | Biochemical | Disk diffusion |
| **Saha 2020** | India | SCBU/premature baby unit | Cross sectional | 2017 | EOS <72hrs | 0-28 days | NR | NR | Phenotypic & biochemical | Disk diffusion |
| **Sana 2018** | Pakistan | NICU | Cross sectional | 2016-2017 | Not defined | NR | NR | NR | NR | Vitek |
| **Sands 2021** | Bangladesh | Multiple inpatient settings | Cohort study | 2015-2017 | EOS <72hrs | 0-60 days | NR | NR | Phenotypic & biochemical | Disk diffusion |
| **Sands 2021** | India | Multiple inpatient settings | Cohort study | 2015-2017 | EOS <72hrs | 0-60 days | NR | NR | Phenotypic & biochemical | Disk diffusion |
| **Sands 2021** | Pakistan | Multiple inpatient settings | Cohort study | 2015-2017 | EOS <72hrs | 0-60 days | NR | NR | Phenotypic & biochemical | Disk diffusion |
| **Shah 2012** | India | NICU | Cross sectional | 2011 | EOS <72hrs | NR | 57/60 (95%) | 42/60 (70%) | NR | Disk diffusion |
| **Shaikh 2020** | Pakistan | NICU | Cross sectional | 2017-2019 | EOS <72hrs | 0-28 days | NR | NR | Phenotypic & biochemical | Disk diffusion |
| **Sharma 2013** | India | NICU | Cross sectional | 2011-2013 | EOS <72hrs | 0-28 days | 77/137 (56%) | 40/137 (29%) | NR | NR |
| **Sheikh 2014** | Pakistan | NICU | Cross sectional | 2013 | EOS <1wk | 0-28 days | 24/40 (60%) | NR | Phenotypic & biochemical | Disk diffusion |
| **Shrestha 2013** | Nepal | NICU | Cross sectional | 2010 | EOS <1wk | NR | NR | 71/99 (72%) | Phenotypic & biochemical | Disk diffusion |
| **Srivastava 2014** | India | NICU | Cross sectional | 2011-2012 | Not defined | NR | NR | NR | Phenotypic & biochemical | NR |
| **Thakur 2016** | India | NICU | Cross sectional | 2012-2013 | Not defined | NR | 92/188 (49%) | 73/188 (39%) | NR | Disk diffusion |
| **Thapa 2013** | Nepal | NICU | Cross sectional | 2011 | Not defined | NR | NR | NR | NR | Disk diffusion |
| **Ullah 2016** | Pakistan | Not stated | Cross sectional | 2012-2015 | Not defined | NR | 1089/1534 (71%) | NR | Phenotypic & biochemical | Disk diffusion |
| **Vaniya 2016** | India | NICU | Cross sectional | 2012-2013 | EOS <1wk | NR | NR | NR | Phenotypic & biochemical | Disk diffusion |
| **Yadav 2018** | Nepal | NICU | Cross sectional | 2015 | EOS <72hrs | 0-28 days | 17/59 (29%) | 22/59 (37%) | Phenotypic & biochemical | Disk diffusion |
| **Africa** | | | | | | | | | | |
| **Abdelaziz 2019** | Sudan | NICU | Cross sectional | 2017-2018 | EOS <1wk | 0-28 days | 130/202 (64%) | NR | Phenotypic & biochemical | Disk diffusion |
| **Almohammady 2020** | Egypt | NICU | Cross sectional | 2017 | EOS <72hrs | NR | 29/70 (41%) | 34/70 (49%) | Phenotypic & biochemical | Disk diffusion |
| **Arowosegbe 2017** | Nigeria | SCBU/premature baby unit | Cross sectional | 2013 | EOS <72hrs | 0-28 days | 13/19 (68%) | NR | Phenotypic & biochemical | Disk diffusion |
| **Ekouya Bowassa 2015** | Congo | Multiple inpatient settings | Cross sectional | 2014 | Not defined | 0-4 days | NR | NR | API | Disk diffusion |
| **El-Amir 2019** | Egypt | NICU | Cross sectional | 2017-2018 | EOS <72hrs | NR | 29/51 (57%) | NR | Phenotypic & biochemical | Disk diffusion |
| **El-Morsi 2020** | Egypt | NICU | Cross sectional | 2012-2015 | EOS <72hrs | 0-28 days | 28/73 (38%) | NR | Phenotypic & biochemical | NR |
| **Gebrehiwot 2012** | Ethiopia | SCBU/premature baby unit | Cross sectional | 2011-2012 | EOS <1wk | 0-28 days | 39/58 (67%) | NR | NR | Disk diffusion |
| **G/Eyesus 2017** | Ethiopia | Hospital | Cross sectional | 2015-2016 | EOS <72hrs | 0-28 days | 84/117 (72%) | 75/117 (64%) | Phenotypic & biochemical | Disk diffusion |
| **Huynh 2018** | Madagascar | Community | Cohort study | 2012-2014 | EOS <1wk | 0-30 days | NR | NR | Phenotypic & biochemical | Disk diffusion |
| **Iregbu 2013** | Nigeria | Multiple inpatient settings | Cross sectional | 2010-2012 | Not defined | NR | NR | NR | Phenotypic & biochemical | Disk diffusion |
| **Iroh 2019** | Malawi | Multiple inpatient settings | Cross sectional | 2008-2017 | EOS <1wk | 0-60 days | NR | NR | Phenotypic & biochemical | Disk diffusion |
| **Kabwe 2016** | Zambia | NICU | Cross sectional | 2013-2014 | EOS <1wk | NR | 88/113 (78%) | NR | Phenotypic & biochemical | Disk diffusion |
| **Labi 2016** | Ghana | Multiple inpatient settings | Cross sectional | 2010-2013 | EOS <48hrs | 0-28 days | 724/1763 (41%) | NR | Phenotypic & biochemical | Disk diffusion |
| **Marando 2018** | Tanzania | NICU | Cross sectional | 2016 | Not defined | 0-28 days | NR | NR | Phenotypic & biochemical | Disk diffusion |
| **Medugu 2017** | Nigeria | Not stated | Cross sectional | 2013-2015 | Not defined | NR | NR | NR | NR | Disk diffusion |
| **Mkony 2014** | Uganda | Paediatric ward | Cross sectional | 2013 | Not defined | 0-28 days | NR | NR | Phenotypic & biochemical | Disk diffusion |
| **Ojide 2013** | Nigeria | Multiple inpatient settings | Cross sectional | 2011-2012 | Not defined | 0-28 days | NR | NR | Phenotypic & biochemical | Disk diffusion |
| **Omoregie 2013** | Nigeria | Paediatric ward | Cross sectional | 2010-2011 | Not defined | 0-28 days | NR | NR | Phenotypic & biochemical | Disk diffusion |
| **Onken 2015** | Tanzania | Multiple inpatient settings | Cross sectional | 2012-2013 | Not defined | 0-30 days | NR | NR | Phenotypic & biochemical | Disk diffusion |
| **Onyedibe 2015** | Nigeria | SCBU/premature baby unit | Cross sectional | 2011 | EOS <72hrs | NR | NR | NR | Phenotypic & biochemical | Disk diffusion |
| **Onyedibe 2016** | Nigeria | SCBU/premature baby unit | Cross sectional | 2011 | Not defined | NR | NR | NR | Phenotypic & biochemical | NR |
| **Pelkonen 2020** | Angola | NICU | Cross sectional | 2016-2017 | EOS <1wk | 0-60 days | NR | NR | Phenotypic & biochemical | Disk diffusion |
| **Peterside 2015** | Nigeria | SCBU/premature baby unit | Cross sectional | 2011-2013 | EOS <72hrs | 0-28 days | 64/97 (66%) | 27/97 (28%) | Phenotypic & biochemical | Disk diffusion |
| **Pius 2016** | Nigeria | SCBU/premature baby unit | Cross sectional | 2012 | EOS <72hrs | 0-28 days | 22/46 (48%) | NR | Phenotypic & biochemical | Disk diffusion |
| **Sands 2021** | Ethiopia | Multiple inpatient settings | Cohort study | 2015-2017 | EOS <72hrs | 0-60 days | NR | NR | Phenotypic & biochemical | Disk diffusion |
| **Sands 2021** | Nigeria | Multiple inpatient settings | Cohort study | 2015-2017 | EOS <72hrs | 0-60 days | NR | NR | Phenotypic & biochemical | Disk diffusion |
| **Sands 2021** | Rwanda | Multiple inpatient settings | Cohort study | 2015-2017 | EOS <72hrs | 0-60 days | NR | NR | Phenotypic & biochemical | Disk diffusion |
| **Shatalov 2015** | Guinea | Not stated | Cross sectional | 2013-2015 | EOS <72hrs | 0-28 days | NR | NR | Phenotypic & biochemical | Disk diffusion |
| **Shehab 2015** | Egypt | NICU | Cross sectional | 2011-2012 | EOS <72hrs | NR | 49/140 (35%) | 91/140 (65%) | Phenotypic & biochemical | Disk diffusion |
| **Shittu 2014** | Nigeria | Paediatric ward | Cross sectional | 2012-2014 | EOS <72hrs | 0-28 days | 63/96 (66%) | NR | Phenotypic & biochemical | Disk diffusion |
| **Shobowale 2017** | Nigeria | NICU | Cross sectional | 2014-2015 | EOS <72hrs | NR | 17/34 (50%) | NR | Phenotypic & biochemical | Disk diffusion |
| **Silago 2020** | Tanzania | NICU | Cross sectional | 2018-2019 | Not defined | 0-28 days | NR | NR | Phenotypic & biochemical | Disk diffusion |
| **Sorsa 2019** | Ethiopia | NICU | Cross sectional | 2016-2017 | EOS <1wk | 0-28 days | 37/88 (42%) | NR | Phenotypic & biochemical | Disk diffusion |

NICU: Neonatal Intensive Care Unit. RCT: Randomised Controlled Trial. NR: Not Reported
