# Supplementary material for: Gram-negative neonatal sepsis in low- and lower-middle-income countries and WHO empirical antibiotic recommendations: A systematic review and meta-analysis
Source: PLoS Med. 2021 Sep 28;18(9):e1003787. doi: 10.1371/journal.pmed.1003787 (PMC8478175; doi:10.1371/journal.pmed.1003787)
Supplement: S1 Text — (DOCX) [file pmed.1003787.s007.docx]

**P** (population/problem): Neonates (<90 days of age) with culture positive Gram-negative sepsis (defined as bacteraemia or infection of otherwise sterile sites).

**I** (intervention): antibiotic treatment with gentamicin, benzylpenicilin/amoxycillin or ceftriaxone (where it is available)

**C** (comparison): not applicable

**O** (outcome): type of Gram-negative pathogens causing neonatal sepsis and the antimicrobial susceptibility pattern

**S** (setting): LMIC

**Database: Pubmed (initial search date 11.03.20, updated search 19.4.21)**


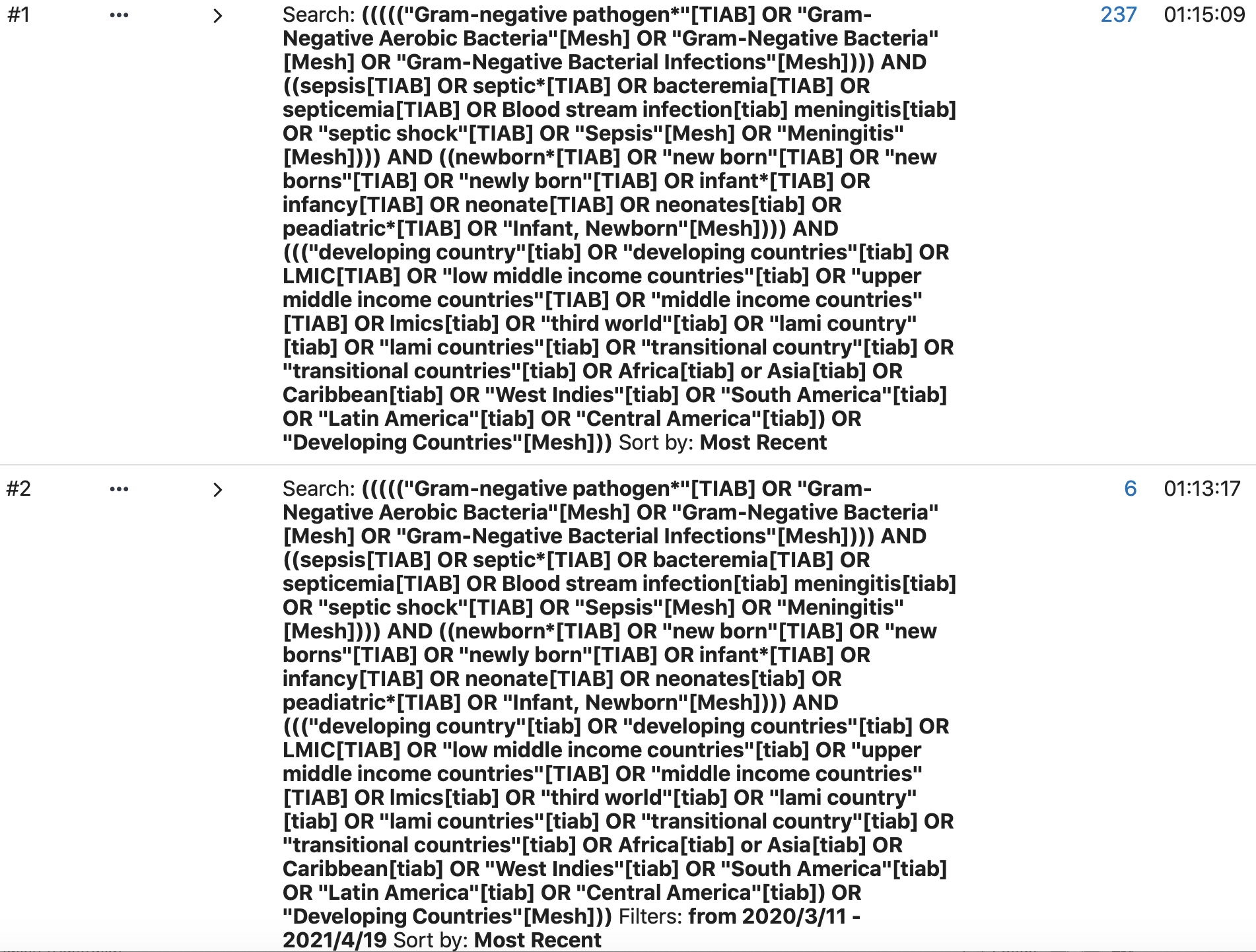


| **Recent queries in pubmed** |  |  |  |
| --- | --- | --- | --- |
| **Search** | Query | Items found | Time |
| **#9** | Search ((((("Gram-negative pathogen*[TIAB]" OR "Gram-Negative Aerobic Bacteria"[Mesh] OR "Gram-Negative Bacteria"[Mesh] OR "Gram-Negative Bacterial Infections"[Mesh]))) AND ((sepsis[TIAB] OR septic*[TIAB] OR bacteremia[TIAB] OR septicemia[TIAB] OR Blood stream infection[tiab] meningitis[tiab] OR "septic shock"[TIAB] OR "Sepsis"[Mesh] OR "Meningitis"[Mesh]))) AND ((newborn*[TIAB] OR "new born"[TIAB] OR "new borns"[TIAB] OR "newly born"[TIAB] OR infant*[TIAB] OR infancy[TIAB] OR neonate[TIAB] OR neonates[tiab] OR peadiatric*[TIAB] OR “Infant, Newborn”[Mesh]))) AND ((("developing country"[tiab] OR "developing countries"[tiab] OR LMIC[TIAB] OR “low middle income countries”[tiab] OR “upper middle income countries”[TIAB] OR “middle income countries”[TIAB] OR lmics[tiab] OR “third world”[tiab] OR “lami country”[tiab] OR “lami countries”[tiab] OR “transitional country”[tiab] OR “transitional countries”[tiab] OR Africa[tiab] or Asia[tiab] OR Caribbean[tiab] OR “West Indies”[tiab] OR “South America”[tiab] OR “Latin America”[tiab] OR “Central America”[tiab]) OR “Developing Countries"[Mesh]))) | 220 | 6:39:56 |
| **#8** | Search (((("Gram-negative pathogen*[TIAB]" OR "Gram-Negative Aerobic Bacteria"[Mesh] OR "Gram-Negative Bacteria"[Mesh] OR "Gram-Negative Bacterial Infections"[Mesh]))) AND ((sepsis[TIAB] OR septic*[TIAB] OR bacteremia[TIAB] OR septicemia[TIAB] OR Blood stream infection[tiab] meningitis[tiab] OR "septic shock"[TIAB] OR "Sepsis"[Mesh] OR "Meningitis"[Mesh]))) AND ((newborn*[TIAB] OR "new born"[TIAB] OR "new borns"[TIAB] OR "newly born"[TIAB] OR infant*[TIAB] OR infancy[TIAB] OR neonate[TIAB] OR neonates[tiab] OR peadiatric*[TIAB] OR “Infant, Newborn”[Mesh])) | 5461 | 6:39:22 |
| **#7** | Search ((("Gram-negative pathogen*[TIAB]" OR "Gram-Negative Aerobic Bacteria"[Mesh] OR "Gram-Negative Bacteria"[Mesh] OR "Gram-Negative Bacterial Infections"[Mesh]))) AND ((sepsis[TIAB] OR septic*[TIAB] OR bacteremia[TIAB] OR septicemia[TIAB] OR Blood stream infection[tiab] meningitis[tiab] OR "septic shock"[TIAB] OR "Sepsis"[Mesh] OR "Meningitis"[Mesh])) | 36749 | 6:39:14 |
| **#6** | Search (("developing country"[tiab] OR "developing countries"[tiab] OR LMIC[TIAB] OR “low middle income countries”[tiab] OR “upper middle income countries”[TIAB] OR “middle income countries”[TIAB] OR lmics[tiab] OR “third world”[tiab] OR “lami country”[tiab] OR “lami countries”[tiab] OR “transitional country”[tiab] OR “transitional countries”[tiab] OR Africa[tiab] or Asia[tiab] OR Caribbean[tiab] OR “West Indies”[tiab] OR “South America”[tiab] OR “Latin America”[tiab] OR “Central America”[tiab]) OR “Developing Countries"[Mesh])) | 315659 | 6:38:57 |
| **#5** | Search (antibiotic OR antimicrobial OR anti-microbial OR antibacterial OR anti-bacterial OR drug) AND (resistance OR resistant*) OR "Drug Resistance"[Mesh]) | 671344 | 6:38:37 |
| **#4** | Search (newborn*[TIAB] OR "new born"[TIAB] OR "new borns"[TIAB] OR "newly born"[TIAB] OR infant*[TIAB] OR infancy[TIAB] OR neonate[TIAB] OR neonates[tiab] OR peadiatric*[TIAB] OR “Infant, Newborn”[Mesh]) | 952542 | 6:38:28 |
| **#3** | Search (sensitivity[TIAB] OR susceptibility[TIAB] OR "Antimicrobial susceptibility" OR "Microbial Sensitivity Tests"[MeSH]) | 1122822 | 6:38:17 |
| **#2** | Search (sepsis[TIAB] OR septic*[TIAB] OR bacteremia[TIAB] OR septicemia[TIAB] OR Blood stream infection[tiab] meningitis[tiab] OR "septic shock"[TIAB] OR "Sepsis"[Mesh] OR "Meningitis"[Mesh]) | 184747 | 6:38:08 |
| **#1** | Search ("Gram-negative pathogen*[TIAB]" OR "Gram-Negative Aerobic Bacteria"[Mesh] OR "Gram-Negative Bacteria"[Mesh] OR "Gram-Negative Bacterial Infections"[Mesh]) | 924839 | 6:37:56 |

**Database: Cochrane (initial search date 01/04/20)**

Date Run: 19/04/2021 21:56:46

Comment:

ID Search Hits

#1 (newborn):ti,ab,kw OR (neonate):ti,ab,kw OR (new born):ti,ab,kw 28214

#2 MeSH descriptor: [Infant, Newborn] explode all trees 16203

#3 #1 OR #2 28332

#4 (sepsis):ti,ab,kw OR (bloodstream infection):ti,ab,kw OR (meningitis):ti,ab,kw OR (bacteraemia):ti,ab,kw 15693

#5 MeSH descriptor: [Neonatal Sepsis] explode all trees 73

#6 MeSH descriptor: [Meninges] explode all trees 295

#7 MeSH descriptor: [Gram-Negative Bacterial Infections] explode all trees 6363

#8 #4 OR #5 OR #6 OR #7 21862

#9 #3 AND #8 1999

#10 (sensitivity):ti,ab,kw OR (susceptibility):ti,ab,kw OR (antimicrobial susceptibility):ti,ab,kw 66345

#11 MeSH descriptor: [Microbial Sensitivity Tests] explode all trees 1477

#12 #10 OR #11 66385

#13 #9 AND #12 107

#14 (antibiotic):ti,ab,kw OR (antimicrobial):ti,ab,kw OR (anti-microbial):ti,ab,kw AND (resistance):ti,ab,kw 27795

#15 MeSH descriptor: [Drug Resistance, Bacterial] explode all trees 892

#16 #14 OR #15 28252

#17 #13 AND #16 40

#18 (developing country):ti,ab,kw OR (developing countries):ti,ab,kw OR (low middle income countries):ti,ab,kw OR (upper middle income countries):ti,ab,kw OR (LMICs):ti,ab,kw 6114

#19 (third world):ti,ab,kw OR (lami country):ti,ab,kw OR (lami countries):ti,ab,kw OR (transitional country):ti,ab,kw OR (transitional countries):ti,ab,kw 1541

#20 (Africa):ti,ab,kw OR (Asia):ti,ab,kw OR (Carribean):ti,ab,kw OR (West Indies):ti,ab,kw 11285

#21 (South America):ti,ab,kw OR (Central America):ti,ab,kw OR (Latin America):ti,ab,kw 1965

#22 MeSH descriptor: [Developing Countries] explode all trees 868

#23 #18 OR #19 OR #20 OR #21 OR #22 19096

#24 #17 AND #23 8

#25 #3 AND #8 AND #23 with Cochrane Library publication date Between Apr 2020 and Apr 2021 10

**Database: Medline (initial search date 08/04/20, updated search date 19/4/21)**

**Database: Embase (initial search date 15/04/20, updated search date 19/4/21)**


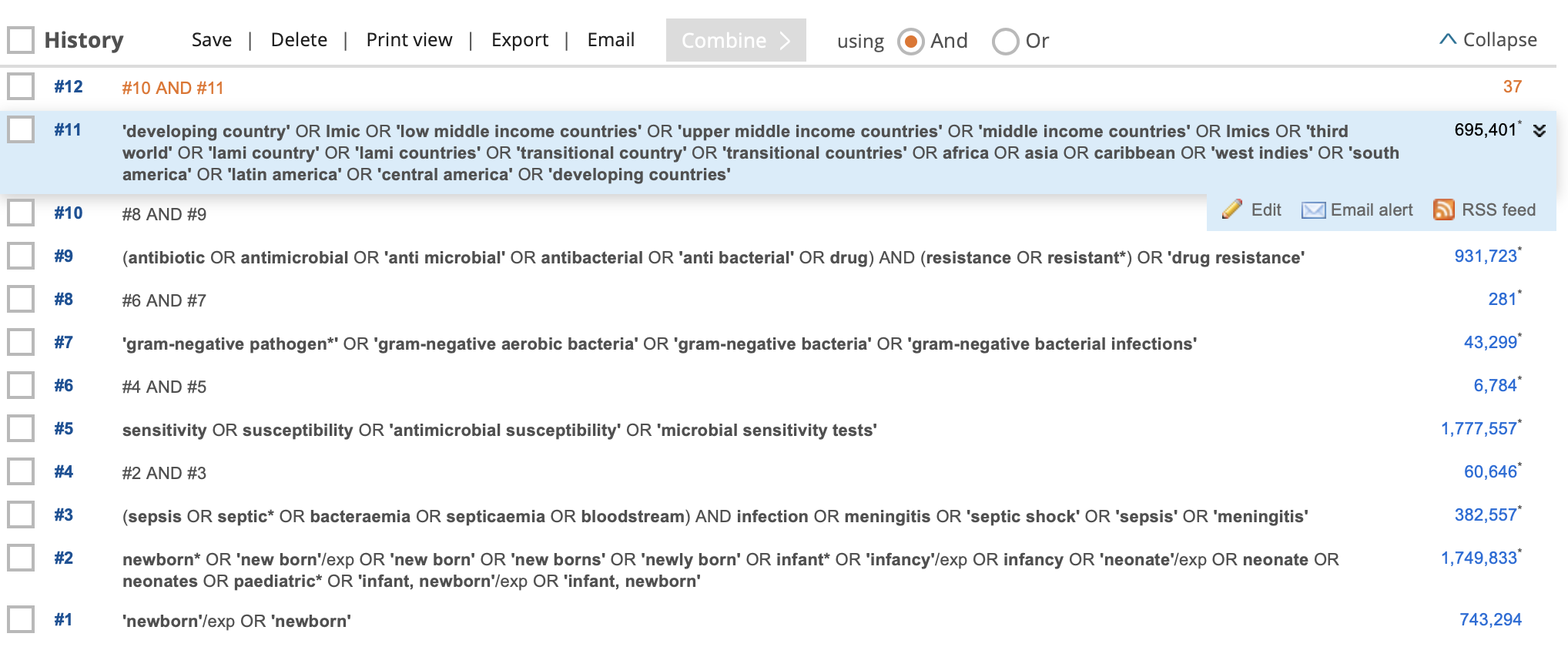


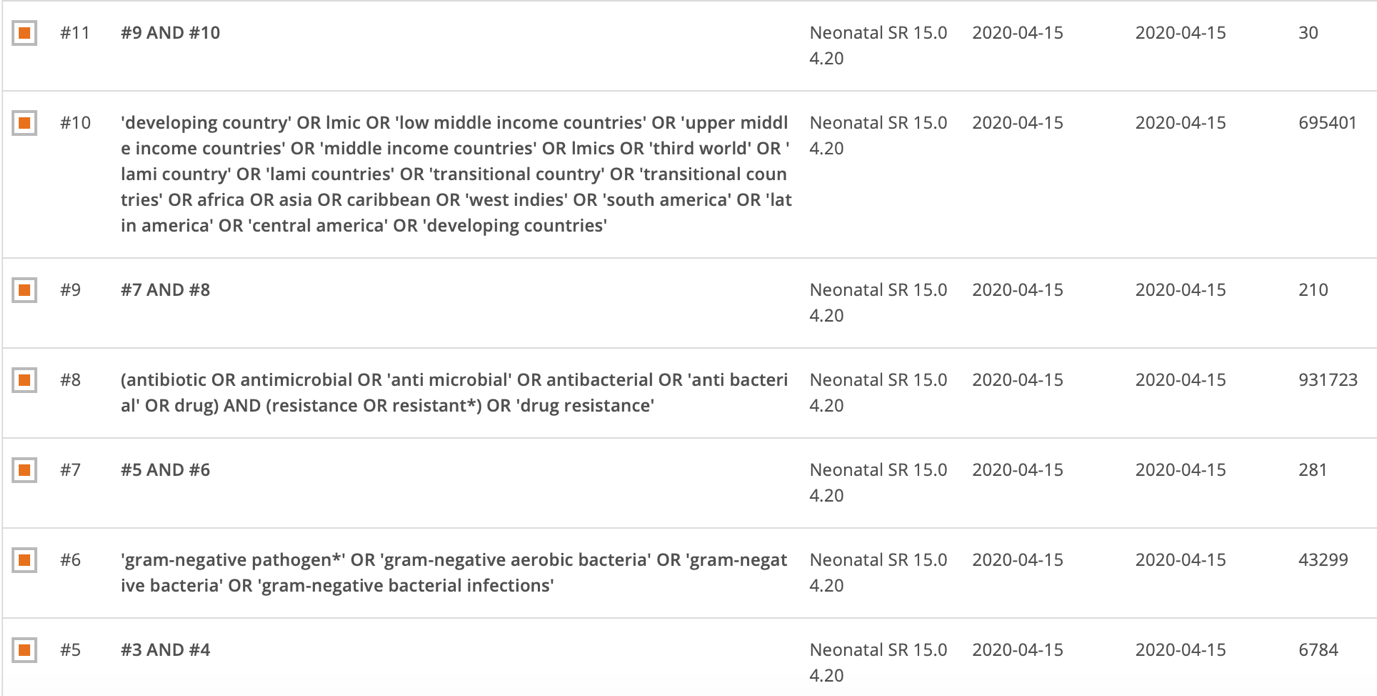


**Database: Web of Science (date of initial search: 15/04/20, updated search date 20/04/21)**


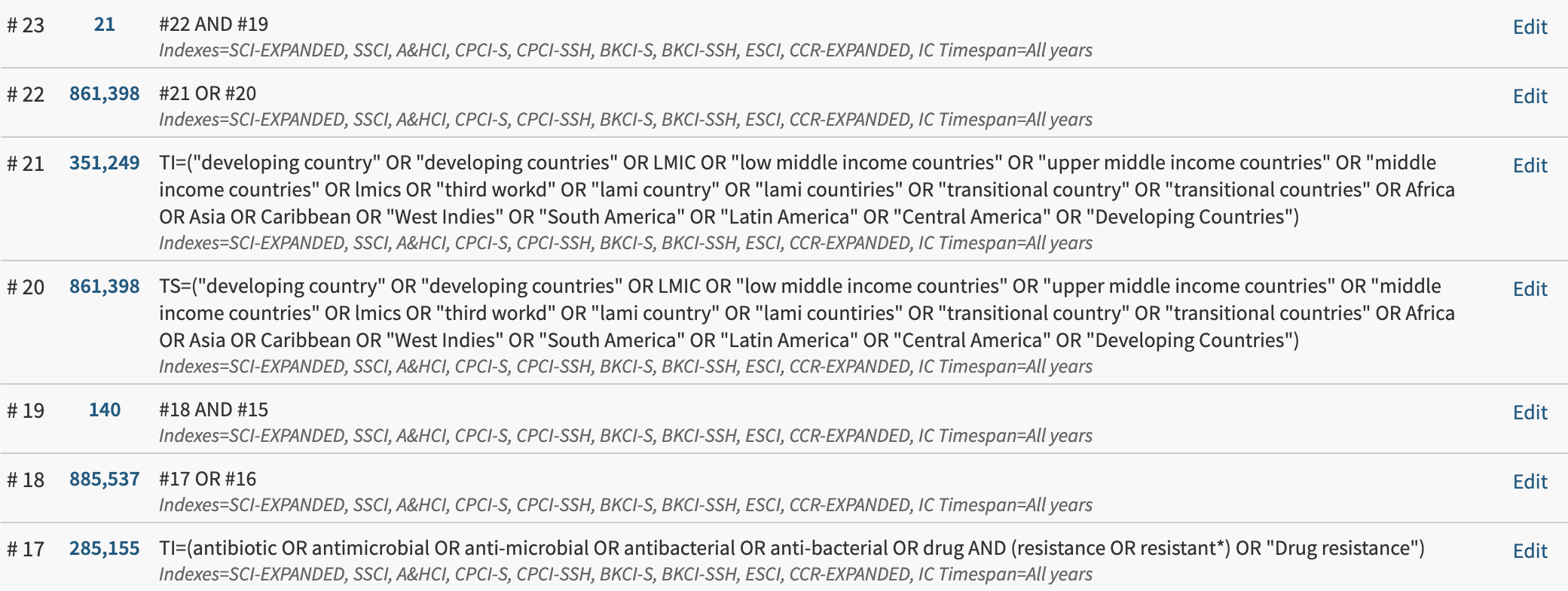


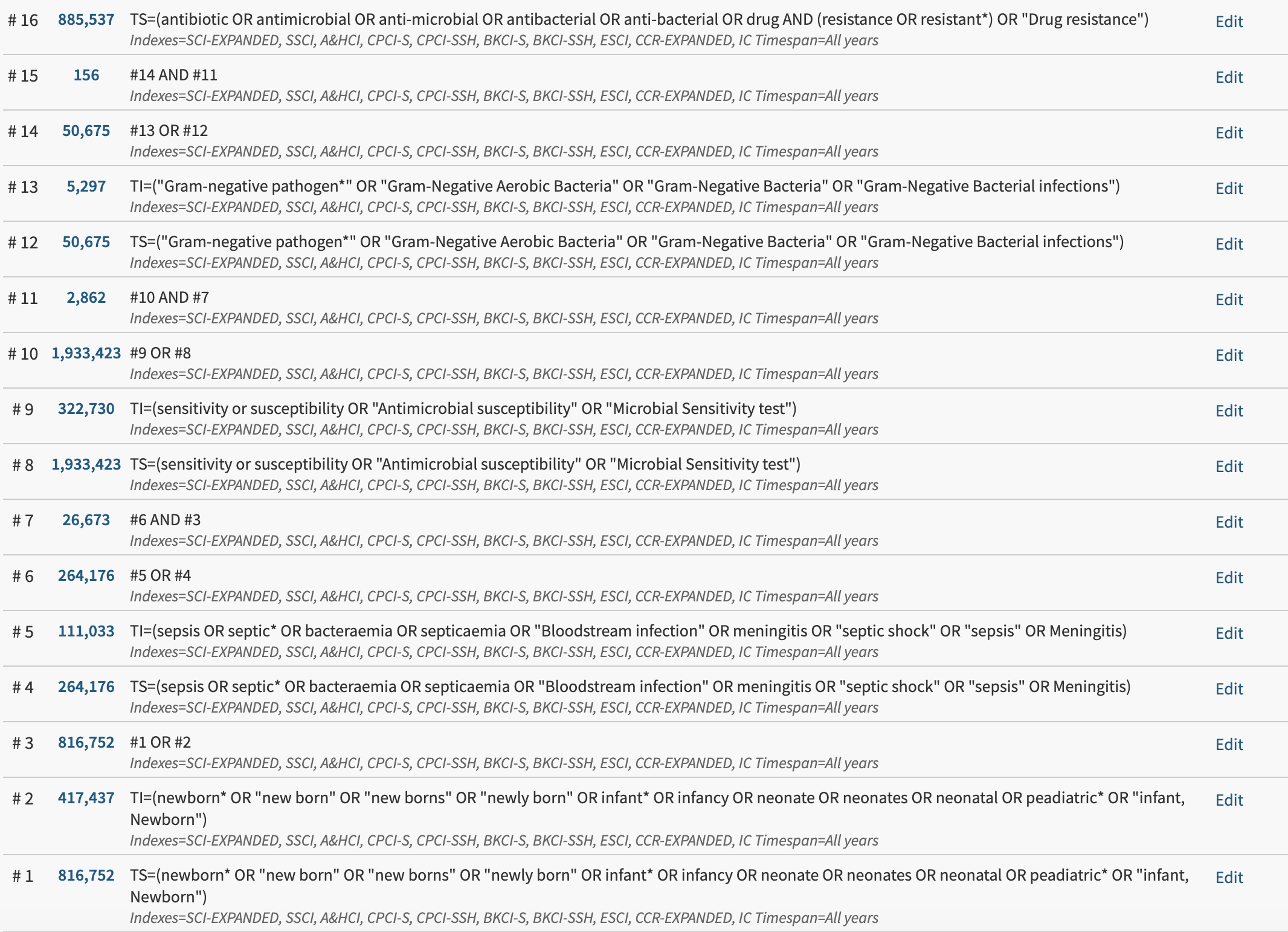


**LILACS (date of search 19/4/21)**

**Google search for grey literature (dates of updated search 21/04/21)**

Search terms used: "newborn sepsis" OR "neonatal sepsis" AND "Gram-negative" AND "multidrug-resistant" OR "resistance"

Site of domain searched: .org and .gov

Region searched : all LMICs as per World Bank group 2019 list.

**Open Grey search (date of search 21/04/21)**

Search terms used: neonatal sepsis, neonatal sepsis AND Gram-negative, newborn infection, pediatric bloodstream infection, pediatric sepsis

**MedCarib (date of search 21/04/21)**

Search terms used: neonatal sepsis, neonatal AND bloodstream AND infection, neonatal AND Gram-negative

**WHOLIS (date of search 21/04/31)**

Search terms used: neonatal sepsis, neonatal AND Gram-negative, neonatal AND bloodstream AND infection, neonatal bacteremia and bacteremia

**WHO Global Index Medicus (date of search 21/04/21)**

Search strategy: tw:((tw:(neonatal sepsis )) OR (tw:(newborn sepsis)) OR (tw:(bloodstream infection))) AND ( collectiongim:("WPRIM") AND mj:("Infant, Newborn" OR "Sepsis" OR "Infant" OR "Intensive Care, Neonatal" OR "Infant, Premature" OR "Anti-Bacterial Agents" OR "Bacteremia" OR "Microbial Sensitivity Tests" OR "Intensive Care Units, Neonatal" OR "Neonatal Sepsis" OR "Epidemiology" OR "Microbiology") AND type_of_study:("incidence_studies" OR "prevalence_studies" OR "clinical_trials" OR "evaluation_studies")) AND (year_cluster:[2010 TO 2021])

Database included: IMEMR, IMSEAR and WPRIM

**African Journal Online (date of search 21/04/21)**

Search terms used: "newborn sepsis" OR "neonatal sepsis" AND "Gram-negative" AND "multidrug-resistant" OR "resistance"
