# Supplementary material for: Gram-negative neonatal sepsis in low- and lower-middle-income countries and WHO empirical antibiotic recommendations: A systematic review and meta-analysis
Source: PLoS Med. 2021 Sep 28;18(9):e1003787. doi: 10.1371/journal.pmed.1003787 (PMC8478175; doi:10.1371/journal.pmed.1003787)
Supplement: Supplementary file 7 [file pmed.1003787.s002.docx]

The Prevalence of *E.coli* among Gram-negative Bacteria

Author	Year of Publication	Country	E.coli isolates	Gram-negative cultures	ES (95% CI)
Africa					
Gebrehiwot	2012	Ethiopia	6	36	0.17 (0.06, 0.33)
Gyawali	2013	Nepal	16	133	0.12 (0.07, 0.19)
Iregbu	2013	Nigeria	1	22	0.05 (0.00, 0.23)
Ojide	2013	Nigeria	23	46	0.50 (0.35, 0.65)
Omoregie	2013	Nigeria	10	142	0.07 (0.03, 0.13)
Mkony	2014	Uganda	12	27	0.44 (0.25, 0.65)
Shittu	2014	Nigeria	18	60	0.30 (0.19, 0.43)
Ekouya Bowassa	2015	Congo	2	18	0.11 (0.01, 0.35)
Onken	2015	Tanzania	6	33	0.18 (0.07, 0.35)
Onyedibe	2015	Nigeria	8	44	0.18 (0.08, 0.33)
Peterside	2015	Nigeria	16	45	0.36 (0.22, 0.51)
Shatalov	2015	Guinea	4	24	0.17 (0.05, 0.37)
Shehab	2015	Egypt	4	54	0.07 (0.02, 0.18)
Kabwe	2016	Zambia	5	84	0.06 (0.02, 0.13)
Labi	2016	Ghana	25	329	0.08 (0.05, 0.11)
Onyedibe	2016	Nigeria	8	39	0.21 (0.09, 0.36)
Pius	2016	Nigeria	9	23	0.39 (0.20, 0.61)
Arowosegbe	2017	Nigeria	1	15	0.07 (0.00, 0.32)
Medugu	2017	Nigeria	3	25	0.12 (0.03, 0.31)
Shobowale	2017	Nigeria	3	20	0.15 (0.03, 0.38)
G/Eyesus.	2017	Ethiopia	12	39	0.31 (0.17, 0.48)
Huynh	2018	Madagascar	2	13	0.15 (0.02, 0.45)
Marando	2018	Tanzania	3	45	0.07 (0.01, 0.18)
Pokhrel	2018	Nepal	3	52	0.06 (0.01, 0.16)
Abdelaziz	2019	Sudan	32	123	0.26 (0.19, 0.35)
El-Amir	2019	Egypt	34	45	0.76 (0.60, 0.87)
Iroh	2019	Malawi	104	722	0.14 (0.12, 0.17)
Sorsa	2019	Ethiopia	18	39	0.46 (0.30, 0.63)
Almohammady	2020	Egypt	0	49	0.00 (0.00, 0.07)
El-Morsi	2020	Egypt	10	60	0.17 (0.08, 0.29)
Hassan	2020	Egypt	19	76	0.25 (0.16, 0.36)
Pelkonen	2020	Angola	17	71	0.24 (0.15, 0.36)
Silago	2020	Tanzania	5	59	0.08 (0.03, 0.19)
Sands-Ethiopia	2021	Ethiopia	11	150	0.07 (0.04, 0.13)
Sands-Nigeria	2021	Nigeria	23	176	0.13 (0.08, 0.19)
Sands-Rwanda	2021	Rwanda	17	80	0.21 (0.13, 0.32)
LR Test: RE vs FE chi^2 = 171.195, p = 0.00)					0.16 (0.12, 0.22)
Asia					
Noor	2012	Indonesia	1	78	0.01 (0.00, 0.07)
Shah	2012	India	12	31	0.39 (0.22, 0.58)
Mehar	2013	India	11	38	0.29 (0.15, 0.46)
Sharma	2013	India	27	76	0.36 (0.25, 0.47)
Shrestha	2013	Nepal	6	57	0.11 (0.04, 0.22)
Thapa	2013	Nepal	0	14	0.00 (0.00, 0.23)
Adhikari	2014	Nepal	25	30	0.83 (0.65, 0.94)
Chelliah	2014	India	14	60	0.23 (0.13, 0.36)
Haque	2014	Bangladesh	2	66	0.03 (0.00, 0.11)
Keerthi	2014	India	6	37	0.16 (0.06, 0.32)
Patel	2014	India	25	196	0.13 (0.08, 0.18)
Sheikh	2014	Pakistan	4	21	0.19 (0.05, 0.42)
Srivastava	2014	India	13	108	0.12 (0.07, 0.20)
Agarwal	2015	India	8	23	0.35 (0.16, 0.57)
Ansari	2015	Nepal	4	42	0.10 (0.03, 0.23)
Dhanawade	2015	India	18	78	0.23 (0.14, 0.34)
Dhanalakshmi	2015	India	4	37	0.11 (0.03, 0.25)
Afrin	2016	Bangladesh	10	13	0.77 (0.46, 0.95)
Agarwal	2016	India	137	640	0.21 (0.18, 0.25)
Bhat B	2016	India	3	60	0.05 (0.01, 0.14)
Lamba	2016	India	19	199	0.10 (0.06, 0.15)
Pandita	2016	India	14	77	0.18 (0.10, 0.29)
Rukmono	2016	Indonesia	1	52	0.02 (0.00, 0.10)
Thakur	2016	India	10	75	0.13 (0.07, 0.23)
Ullah	2016	Pakistan	811	1206	0.67 (0.65, 0.70)
Vaniya	2016	India	24	209	0.11 (0.07, 0.17)
Dalal	2017	India	44	289	0.15 (0.11, 0.20)
Kanodia	2017	Nepal	11	96	0.11 (0.06, 0.20)
Kumar DVP	2017	India	3	17	0.18 (0.04, 0.43)
Mir	2017	Pakistan	3	43	0.07 (0.01, 0.19)
Rohatgi	2017	India	0	85	0.00 (0.00, 0.04)
Roy M	2017	India	230	1161	0.20 (0.18, 0.22)
Ahmed	2018	Pakistan	119	202	0.59 (0.52, 0.66)
Ahmed F	2018	Bangladesh	0	58	0.00 (0.00, 0.06)
Annapurna	2018	India	21	94	0.22 (0.14, 0.32)
Bandyopadhyay	2018	India	12	80	0.15 (0.08, 0.25)
Dhaneria	2018	India	5	26	0.19 (0.07, 0.39)
Garg	2018	India	3	14	0.21 (0.05, 0.51)
Haider	2018	India	11	77	0.14 (0.07, 0.24)
Jajoo	2018	India	32	170	0.19 (0.13, 0.26)
Mishra	2018	India	30	73	0.41 (0.30, 0.53)
Sana	2018	Pakistan	3	75	0.04 (0.01, 0.11)
Yadav	2018	Nepal	4	32	0.12 (0.04, 0.29)
Mitra	2019	India	16	73	0.22 (0.13, 0.33)
Mukherjee P	2019	India	10	51	0.20 (0.10, 0.33)
Mukherjee S	2019	India	4	200	0.02 (0.01, 0.05)
Bhargava	2020	Nepal	2	19	0.11 (0.01, 0.33)
Ghosh	2020	India	10	32	0.31 (0.16, 0.50)
Govindaraju	2020	India	5	47	0.11 (0.04, 0.23)
Saha	2020	India	9	27	0.33 (0.17, 0.54)
Shaikh	2020	Pakistan	11	79	0.14 (0.07, 0.24)
Sands-Bangladesh	2021	Bangladesh	3	184	0.02 (0.00, 0.05)
Sands-India	2021	India	2	14	0.14 (0.02, 0.43)
Sands-Pakistan	2021	Pakistan	13	395	0.03 (0.02, 0.06)
LR Test: RE vs FE chi^2 = 1673.523, p = 0.00)					0.14 (0.10, 0.19)
Heterogeneity between groups: p = 0.434					
LR Test: RE vs FE chi^2 = 1945.49, p = 0.00);					0.15 (0.12, 0.18)

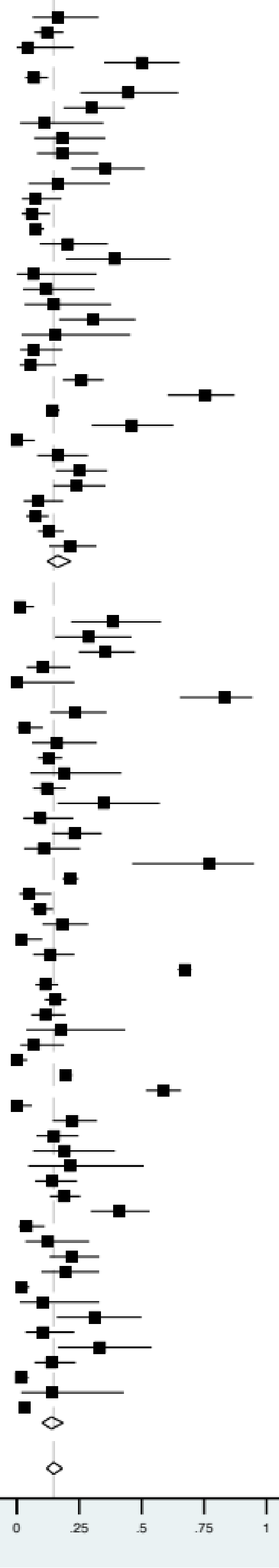

The Prevalence of *Klebsiella* spp among Gram-negative Bacteria

Author	Year of Publication	Country	Klebsiella spp isolates	Gram-negative cultures	ES (95% CI)
Africa					
Gebrehiwot	2012	Ethiopia	16	36	0.44 (0.28, 0.62)
Gyawali	2013	Nepal	32	133	0.24 (0.17, 0.32)
Iregbu	2013	Nigeria	9	22	0.41 (0.21, 0.64)
Ojide	2013	Nigeria	15	46	0.33 (0.20, 0.48)
Omoregie	2013	Nigeria	74	142	0.52 (0.44, 0.61)
Mkony	2014	Uganda	14	27	0.52 (0.32, 0.71)
Shittu	2014	Nigeria	33	60	0.55 (0.42, 0.68)
Ekouya Bowassa	2015	Congo	12	18	0.67 (0.41, 0.87)
Onken	2015	Tanzania	8	33	0.24 (0.11, 0.42)
Onyedibe	2015	Nigeria	24	44	0.55 (0.39, 0.70)
Peterside	2015	Nigeria	14	45	0.31 (0.18, 0.47)
Shatalov	2015	Guinea	16	24	0.67 (0.45, 0.84)
Shehab	2015	Egypt	20	54	0.37 (0.24, 0.51)
Kabwe	2016	Zambia	77	84	0.92 (0.84, 0.97)
Labi	2016	Ghana	31	329	0.09 (0.06, 0.13)
Onyedibe	2016	Nigeria	22	39	0.56 (0.40, 0.72)
Pius	2016	Nigeria	7	23	0.30 (0.13, 0.53)
Arowosegbe	2017	Nigeria	6	15	0.40 (0.16, 0.68)
Medugu	2017	Nigeria	9	25	0.36 (0.18, 0.57)
Shobowale	2017	Nigeria	8	20	0.40 (0.19, 0.64)
G/Eyesus.	2017	Ethiopia	19	39	0.49 (0.32, 0.65)
Huynh	2018	Madagascar	6	13	0.46 (0.19, 0.75)
Marando	2018	Tanzania	26	45	0.58 (0.42, 0.72)
Pokhrel	2018	Nepal	23	52	0.44 (0.30, 0.59)
Abdelaziz	2019	Sudan	43	123	0.35 (0.27, 0.44)
El-Amir	2019	Egypt	6	45	0.13 (0.05, 0.27)
Iroh	2019	Malawi	307	722	0.43 (0.39, 0.46)
Sorsa	2019	Ethiopia	11	39	0.28 (0.15, 0.45)
Almohammady	2020	Egypt	34	49	0.69 (0.55, 0.82)
El-Morsi	2020	Egypt	43	60	0.72 (0.59, 0.83)
Hassan	2020	Egypt	27	76	0.36 (0.25, 0.47)
Pelkonen	2020	Angola	30	71	0.42 (0.31, 0.55)
Silago	2020	Tanzania	28	59	0.47 (0.34, 0.61)
Sands-Ethiopia	2021	Ethiopia	101	150	0.67 (0.59, 0.75)
Sands-Nigeria	2021	Nigeria	63	176	0.36 (0.29, 0.43)
Sands-Rwanda	2021	Rwanda	27	80	0.34 (0.24, 0.45)
LR Test: RE vs FE chi^2 = 326.014, p = 0.00)					0.44 (0.37, 0.50)
Asia					
Noor	2012	Indonesia	23	78	0.29 (0.20, 0.41)
Shah	2012	India	7	31	0.23 (0.10, 0.41)
Mehar	2013	India	13	38	0.34 (0.20, 0.51)
Sharma	2013	India	37	76	0.49 (0.37, 0.60)
Shrestha	2013	Nepal	27	57	0.47 (0.34, 0.61)
Thapa	2013	Nepal	4	14	0.29 (0.08, 0.58)
Adhikari	2014	Nepal	4	30	0.13 (0.04, 0.31)
Chelliah	2014	India	35	60	0.58 (0.45, 0.71)
Haque	2014	Bangladesh	6	66	0.09 (0.03, 0.19)
Keerthi	2014	India	20	37	0.54 (0.37, 0.71)
Patel	2014	India	117	196	0.60 (0.52, 0.67)
Sheikh	2014	Pakistan	6	21	0.29 (0.11, 0.52)
Srivastava	2014	India	25	108	0.23 (0.16, 0.32)
Agarwal	2015	India	9	23	0.39 (0.20, 0.61)
Ansari	2015	Nepal	9	42	0.21 (0.10, 0.37)
Dhanawade	2015	India	37	78	0.47 (0.36, 0.59)
Dhanalakshmi	2015	India	28	37	0.76 (0.59, 0.88)
Afrin	2016	Bangladesh	2	13	0.15 (0.02, 0.45)
Agarwal	2016	India	169	640	0.26 (0.23, 0.30)
Bhat B	2016	India	39	60	0.65 (0.52, 0.77)
Lamba	2016	India	49	199	0.25 (0.19, 0.31)
Pandita	2016	India	33	77	0.43 (0.32, 0.55)
Rukmono	2016	Indonesia	6	52	0.12 (0.04, 0.23)
Thakur	2016	India	18	75	0.24 (0.15, 0.35)
Ullah	2016	Pakistan	102	1206	0.08 (0.07, 0.10)
Vaniya	2016	India	154	209	0.74 (0.67, 0.80)
Dalal	2017	India	15	289	0.05 (0.03, 0.08)
Kanodia	2017	Nepal	8	96	0.08 (0.04, 0.16)
Kumar DVP	2017	India	6	17	0.35 (0.14, 0.62)
Mir	2017	Pakistan	2	43	0.05 (0.01, 0.16)
Rohatgi	2017	India	54	85	0.64 (0.52, 0.74)
Roy M	2017	India	242	1161	0.21 (0.19, 0.23)
Ahmed	2018	Pakistan	43	202	0.21 (0.16, 0.28)
Ahmed F	2018	Bangladesh	14	58	0.24 (0.14, 0.37)
Annapurna	2018	India	41	94	0.44 (0.33, 0.54)
Bandyopadhyay	2018	India	57	80	0.71 (0.60, 0.81)
Dhaneria	2018	India	11	26	0.42 (0.23, 0.63)
Garg	2018	India	5	14	0.36 (0.13, 0.65)
Haider	2018	India	35	77	0.45 (0.34, 0.57)
Jajoo	2018	India	55	170	0.32 (0.25, 0.40)
Mishra	2018	India	23	73	0.32 (0.21, 0.43)
Sana	2018	Pakistan	24	75	0.32 (0.22, 0.44)
Yadav	2018	Nepal	9	32	0.28 (0.14, 0.47)
Mitra	2019	India	55	73	0.75 (0.64, 0.85)
Mukherjee P	2019	India	18	51	0.35 (0.22, 0.50)
Mukherjee S	2019	India	184	200	0.92 (0.87, 0.95)
Bhargava	2020	Nepal	10	19	0.53 (0.29, 0.76)
Ghosh	2020	India	17	32	0.53 (0.35, 0.71)
Govindaraju	2020	India	23	47	0.49 (0.34, 0.64)
Saha	2020	India	18	27	0.67 (0.46, 0.83)
Shaikh	2020	Pakistan	17	79	0.22 (0.13, 0.32)
Sands-Bangladesh	2021	Bangladesh	27	184	0.15 (0.10, 0.21)
Sands-India	2021	India	5	14	0.36 (0.13, 0.65)
Sands-Pakistan	2021	Pakistan	161	395	0.41 (0.36, 0.46)
LR Test: RE vs FE chi^2 = 1379.354, p = 0.00)					0.35 (0.29, 0.42)
Heterogeneity between groups: p = 0.073					
LR Test: RE vs FE chi^2 = 1820.26, p = 0.00);					0.38 (0.33, 0.43)

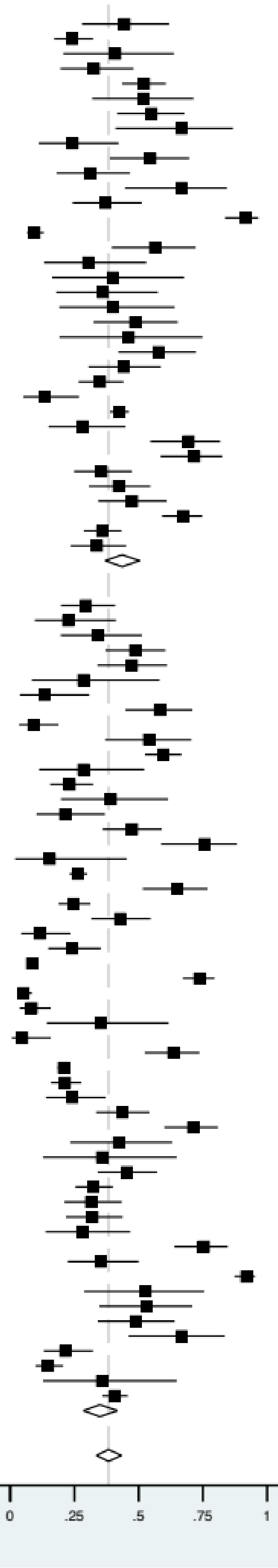

The Prevalence of *Enterobacter* spp among Gram-negative Bacteria

Author	Year of Publication	Country	Enterobacter spp isolates	Gram-negative cultures	ES (95% CI)
Africa					
Gebrehiwot	2012	Ethiopia	1	36	0.03 (0.00, 0.15)
Gyawali	2013	Nepal	27	133	0.20 (0.14, 0.28)
Iregbu	2013	Nigeria	0	22	0.00 (0.00, 0.15)
Ojide	2013	Nigeria	5	46	0.11 (0.04, 0.24)
Omoregie	2013	Nigeria	5	142	0.04 (0.01, 0.08)
Mkony	2014	Uganda	0	27	0.00 (0.00, 0.13)
Shittu	2014	Nigeria	0	60	0.00 (0.00, 0.06)
Ekouya Bowassa	2015	Congo	4	18	0.22 (0.06, 0.48)
Onyedibe	2015	Nigeria	2	44	0.05 (0.01, 0.15)
Peterside	2015	Nigeria	0	45	0.00 (0.00, 0.08)
Shatalov	2015	Guinea	0	24	0.00 (0.00, 0.14)
Shehab	2015	Egypt	1	54	0.02 (0.00, 0.10)
Kabwe	2016	Zambia	0	84	0.00 (0.00, 0.04)
Labi	2016	Ghana	59	329	0.18 (0.14, 0.23)
Onyedibe	2016	Nigeria	2	39	0.05 (0.01, 0.17)
Arowosegbe	2017	Nigeria	4	15	0.27 (0.08, 0.55)
Medugu	2017	Nigeria	3	25	0.12 (0.03, 0.31)
G/Eyesus.	2017	Ethiopia	0	39	0.00 (0.00, 0.09)
Huynh	2018	Madagascar	1	13	0.08 (0.00, 0.36)
Marando	2018	Tanzania	2	45	0.04 (0.01, 0.15)
Pokhrel	2018	Nepal	13	52	0.25 (0.14, 0.39)
Abdelaziz	2019	Sudan	6	123	0.05 (0.02, 0.10)
El-Amir	2019	Egypt	0	45	0.00 (0.00, 0.08)
Iroh	2019	Malawi	118	722	0.16 (0.14, 0.19)
Sorsa	2019	Ethiopia	7	39	0.18 (0.08, 0.34)
Almohammady	2020	Egypt	3	49	0.06 (0.01, 0.17)
El-Morsi	2020	Egypt	0	60	0.00 (0.00, 0.06)
Hassan	2020	Egypt	3	76	0.04 (0.01, 0.11)
Pelkonen	2020	Angola	7	71	0.10 (0.04, 0.19)
Silago	2020	Tanzania	2	59	0.03 (0.00, 0.12)
Sands-Ethiopia	2021	Ethiopia	0	150	0.00 (0.00, 0.02)
Sands-Nigeria	2021	Nigeria	19	176	0.11 (0.07, 0.16)
Sands-Rwanda	2021	Rwanda	19	80	0.24 (0.15, 0.35)
LR Test: RE vs FE chi^2 = 144.714, p = 0.00)					0.04 (0.02, 0.08)
Asia					
Noor	2012	Indonesia	5	78	0.06 (0.02, 0.14)
Shah	2012	India	0	31	0.00 (0.00, 0.11)
Mehar	2013	India	4	38	0.11 (0.03, 0.25)
Sharma	2013	India	3	76	0.04 (0.01, 0.11)
Shrestha	2013	Nepal	10	57	0.18 (0.09, 0.30)
Thapa	2013	Nepal	0	14	0.00 (0.00, 0.23)
Adhikari	2014	Nepal	0	30	0.00 (0.00, 0.12)
Chelliah	2014	India	0	60	0.00 (0.00, 0.06)
Haque	2014	Bangladesh	3	66	0.05 (0.01, 0.13)
Keerthi	2014	India	1	37	0.03 (0.00, 0.14)
Patel	2014	India	26	196	0.13 (0.09, 0.19)
Sheikh	2014	Pakistan	3	21	0.14 (0.03, 0.36)
Srivastava	2014	India	2	108	0.02 (0.00, 0.07)
Agarwal	2015	India	1	23	0.04 (0.00, 0.22)
Ansari	2015	Nepal	6	42	0.14 (0.05, 0.29)
Dhanawade	2015	India	0	78	0.00 (0.00, 0.05)
Dhanalakshmi	2015	India	0	37	0.00 (0.00, 0.09)
Afrin	2016	Bangladesh	0	13	0.00 (0.00, 0.25)
Agarwal	2016	India	44	640	0.07 (0.05, 0.09)
Bhat B	2016	India	1	60	0.02 (0.00, 0.09)
Lamba	2016	India	56	199	0.28 (0.22, 0.35)
Pandita	2016	India	7	77	0.09 (0.04, 0.18)
Rukmono	2016	Indonesia	3	52	0.06 (0.01, 0.16)
Thakur	2016	India	10	75	0.13 (0.07, 0.23)
Ullah	2016	Pakistan	0	1206	0.00 (0.00, 0.00)
Vaniya	2016	India	0	209	0.00 (0.00, 0.02)
Dalal	2017	India	2	289	0.01 (0.00, 0.02)
Kanodia	2017	Nepal	10	96	0.10 (0.05, 0.18)
Mir	2017	Pakistan	1	43	0.02 (0.00, 0.12)
Rohatgi	2017	India	22	85	0.26 (0.17, 0.37)
Ahmed	2018	Pakistan	28	202	0.14 (0.09, 0.19)
Ahmed F	2018	Bangladesh	6	58	0.10 (0.04, 0.21)
Annapurna	2018	India	0	94	0.00 (0.00, 0.04)
Bandyopadhyay	2018	India	0	80	0.00 (0.00, 0.05)
Garg	2018	India	0	14	0.00 (0.00, 0.23)
Haider	2018	India	8	77	0.10 (0.05, 0.19)
Jajoo	2018	India	23	170	0.14 (0.09, 0.20)
Mishra	2018	India	0	73	0.00 (0.00, 0.05)
Sana	2018	Pakistan	2	75	0.03 (0.00, 0.09)
Yadav	2018	Nepal	6	32	0.19 (0.07, 0.36)
Mitra	2019	India	2	73	0.03 (0.00, 0.10)
Mukherjee P	2019	India	0	51	0.00 (0.00, 0.07)
Mukherjee S	2019	India	6	200	0.03 (0.01, 0.06)
Bhargava	2020	Nepal	0	19	0.00 (0.00, 0.18)
Ghosh	2020	India	0	32	0.00 (0.00, 0.11)
Govindaraju	2020	India	3	47	0.06 (0.01, 0.18)
Saha	2020	India	0	27	0.00 (0.00, 0.13)
Shaikh	2020	Pakistan	0	79	0.00 (0.00, 0.05)
Sands-Bangladesh	2021	Bangladesh	2	184	0.01 (0.00, 0.04)
Sands-India	2021	India	0	14	0.00 (0.00, 0.23)
Sands-Pakistan	2021	Pakistan	39	395	0.10 (0.07, 0.13)
LR Test: RE vs FE chi^2 = 389.216, p = 0.00)					0.03 (0.01, 0.04)
Heterogeneity between groups: p = 0.210					
LR Test: RE vs FE chi^2 = 599.07, p = 0.00);					0.03 (0.02, 0.05)

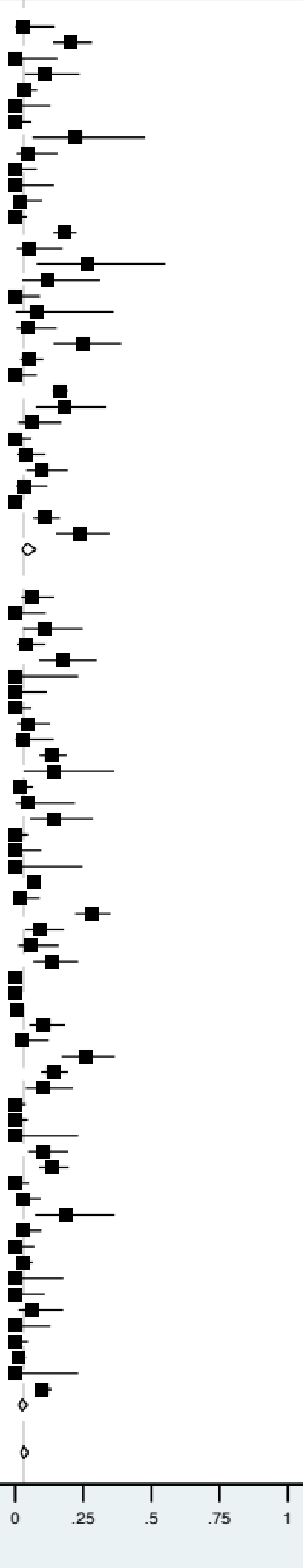

The Prevalence of *Pseudomonas* spp among Gram-negative Bacteria

Author	Year of Publication	Country	Pseudomonas spp isolates	Gram-negative cultures	ES (95% CI)
Africa					
Gebrehiwot	2012	Ethiopia	0	36	0.00 (0.00, 0.10)
Gyawali	2013	Nepal	21	133	0.16 (0.10, 0.23)
Iregbu	2013	Nigeria	5	22	0.23 (0.08, 0.45)
Ojide	2013	Nigeria	0	46	0.00 (0.00, 0.08)
Omoregie	2013	Nigeria	8	142	0.06 (0.02, 0.11)
Mkony	2014	Uganda	1	27	0.04 (0.00, 0.19)
Shittu	2014	Nigeria	6	60	0.10 (0.04, 0.21)
Ekouya Bowassa	2015	Congo	0	18	0.00 (0.00, 0.19)
Onyedibe	2015	Nigeria	3	44	0.07 (0.01, 0.19)
Peterside	2015	Nigeria	7	45	0.16 (0.06, 0.29)
Shatalov	2015	Guinea	0	24	0.00 (0.00, 0.14)
Shehab	2015	Egypt	2	54	0.04 (0.00, 0.13)
Kabwe	2016	Zambia	0	84	0.00 (0.00, 0.04)
Labi	2016	Ghana	27	329	0.08 (0.05, 0.12)
Onyedibe	2016	Nigeria	1	39	0.03 (0.00, 0.13)
Arowosegbe	2017	Nigeria	2	15	0.13 (0.02, 0.40)
Medugu	2017	Nigeria	7	25	0.28 (0.12, 0.49)
Shobowale	2017	Nigeria	6	20	0.30 (0.12, 0.54)
G/Eyesus.	2017	Ethiopia	0	39	0.00 (0.00, 0.09)
Huynh	2018	Madagascar	0	13	0.00 (0.00, 0.25)
Marando	2018	Tanzania	2	45	0.04 (0.01, 0.15)
Pokhrel	2018	Nepal	2	52	0.04 (0.00, 0.13)
Abdelaziz	2019	Sudan	18	123	0.15 (0.09, 0.22)
El-Amir	2019	Egypt	3	45	0.07 (0.01, 0.18)
Iroh	2019	Malawi	42	722	0.06 (0.04, 0.08)
Sorsa	2019	Ethiopia	0	39	0.00 (0.00, 0.09)
Almohammady	2020	Egypt	4	49	0.08 (0.02, 0.20)
El-Morsi	2020	Egypt	5	60	0.08 (0.03, 0.18)
Hassan	2020	Egypt	6	76	0.08 (0.03, 0.16)
Pelkonen	2020	Angola	7	71	0.10 (0.04, 0.19)
Silago	2020	Tanzania	0	59	0.00 (0.00, 0.06)
Sands-Ethiopia	2021	Ethiopia	1	150	0.01 (0.00, 0.04)
Sands-Nigeria	2021	Nigeria	12	176	0.07 (0.04, 0.12)
Sands-Rwanda	2021	Rwanda	2	80	0.03 (0.00, 0.09)
LR Test: RE vs FE chi^2 = 40.659, p = 0.00)					0.05 (0.03, 0.08)
Asia					
Noor	2012	Indonesia	1	78	0.01 (0.00, 0.07)
Shah	2012	India	6	31	0.19 (0.07, 0.37)
Mehar	2013	India	4	38	0.11 (0.03, 0.25)
Sharma	2013	India	6	76	0.08 (0.03, 0.16)
Shrestha	2013	Nepal	9	57	0.16 (0.07, 0.28)
Thapa	2013	Nepal	5	14	0.36 (0.13, 0.65)
Adhikari	2014	Nepal	1	30	0.03 (0.00, 0.17)
Chelliah	2014	India	11	60	0.18 (0.10, 0.30)
Haque	2014	Bangladesh	19	66	0.29 (0.18, 0.41)
Keerthi	2014	India	3	37	0.08 (0.02, 0.22)
Patel	2014	India	15	196	0.08 (0.04, 0.12)
Sheikh	2014	Pakistan	8	21	0.38 (0.18, 0.62)
Srivastava	2014	India	18	108	0.17 (0.10, 0.25)
Agarwal	2015	India	0	23	0.00 (0.00, 0.15)
Ansari	2015	Nepal	6	42	0.14 (0.05, 0.29)
Dhanawade	2015	India	0	78	0.00 (0.00, 0.05)
Dhanalakshmi	2015	India	3	37	0.08 (0.02, 0.22)
Afrin	2016	Bangladesh	1	13	0.08 (0.00, 0.36)
Agarwal	2016	India	68	640	0.11 (0.08, 0.13)
Bhat B	2016	India	1	60	0.02 (0.00, 0.09)
Chatterjee	2016	India	0	68	0.00 (0.00, 0.05)
Lamba	2016	India	29	199	0.15 (0.10, 0.20)
Pandita	2016	India	4	77	0.05 (0.01, 0.13)
Rukmono	2016	Indonesia	23	52	0.44 (0.30, 0.59)
Ullah	2016	Pakistan	199	1206	0.17 (0.14, 0.19)
Vaniya	2016	India	9	209	0.04 (0.02, 0.08)
Dalal	2017	India	169	289	0.58 (0.53, 0.64)
Kanodia	2017	Nepal	18	96	0.19 (0.12, 0.28)
Kumar DVP	2017	India	1	17	0.06 (0.00, 0.29)
Mir	2017	Pakistan	5	43	0.12 (0.04, 0.25)
Rohatgi	2017	India	1	85	0.01 (0.00, 0.06)
Ahmed	2018	Pakistan	9	202	0.04 (0.02, 0.08)
Ahmed F	2018	Bangladesh	1	58	0.02 (0.00, 0.09)
Annapurna	2018	India	20	94	0.21 (0.14, 0.31)
Bandyopadhyay	2018	India	2	80	0.03 (0.00, 0.09)
Dhaneria	2018	India	6	26	0.23 (0.09, 0.44)
Garg	2018	India	2	14	0.14 (0.02, 0.43)
Haider	2018	India	6	77	0.08 (0.03, 0.16)
Jajoo	2018	India	1	170	0.01 (0.00, 0.03)
Mishra	2018	India	8	73	0.11 (0.05, 0.20)
Yadav	2018	Nepal	4	32	0.12 (0.04, 0.29)
Mukherjee P	2019	India	5	51	0.10 (0.03, 0.21)
Bhargava	2020	Nepal	6	19	0.32 (0.13, 0.57)
Ghosh	2020	India	3	32	0.09 (0.02, 0.25)
Govindaraju	2020	India	7	47	0.15 (0.06, 0.28)
Saha	2020	India	0	27	0.00 (0.00, 0.13)
Shaikh	2020	Pakistan	42	79	0.53 (0.42, 0.64)
Sands-Bangladesh	2021	Bangladesh	6	184	0.03 (0.01, 0.07)
Sands-India	2021	India	1	14	0.07 (0.00, 0.34)
Sands-Pakistan	2021	Pakistan	14	395	0.04 (0.02, 0.06)
LR Test: RE vs FE chi^2 = 608.049, p = 0.00)					0.09 (0.06, 0.13)
Heterogeneity between groups: p = 0.033					
LR Test: RE vs FE chi^2 = 745.20, p = 0.00);					0.07 (0.05, 0.09)

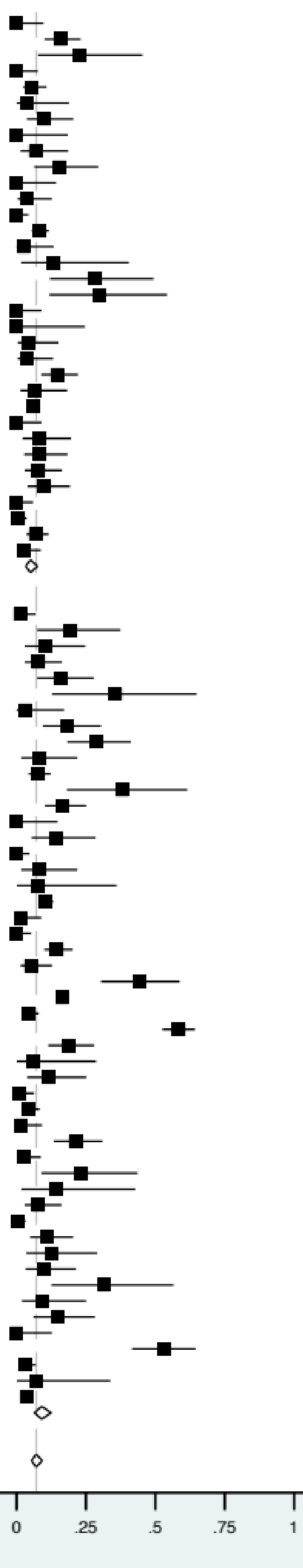

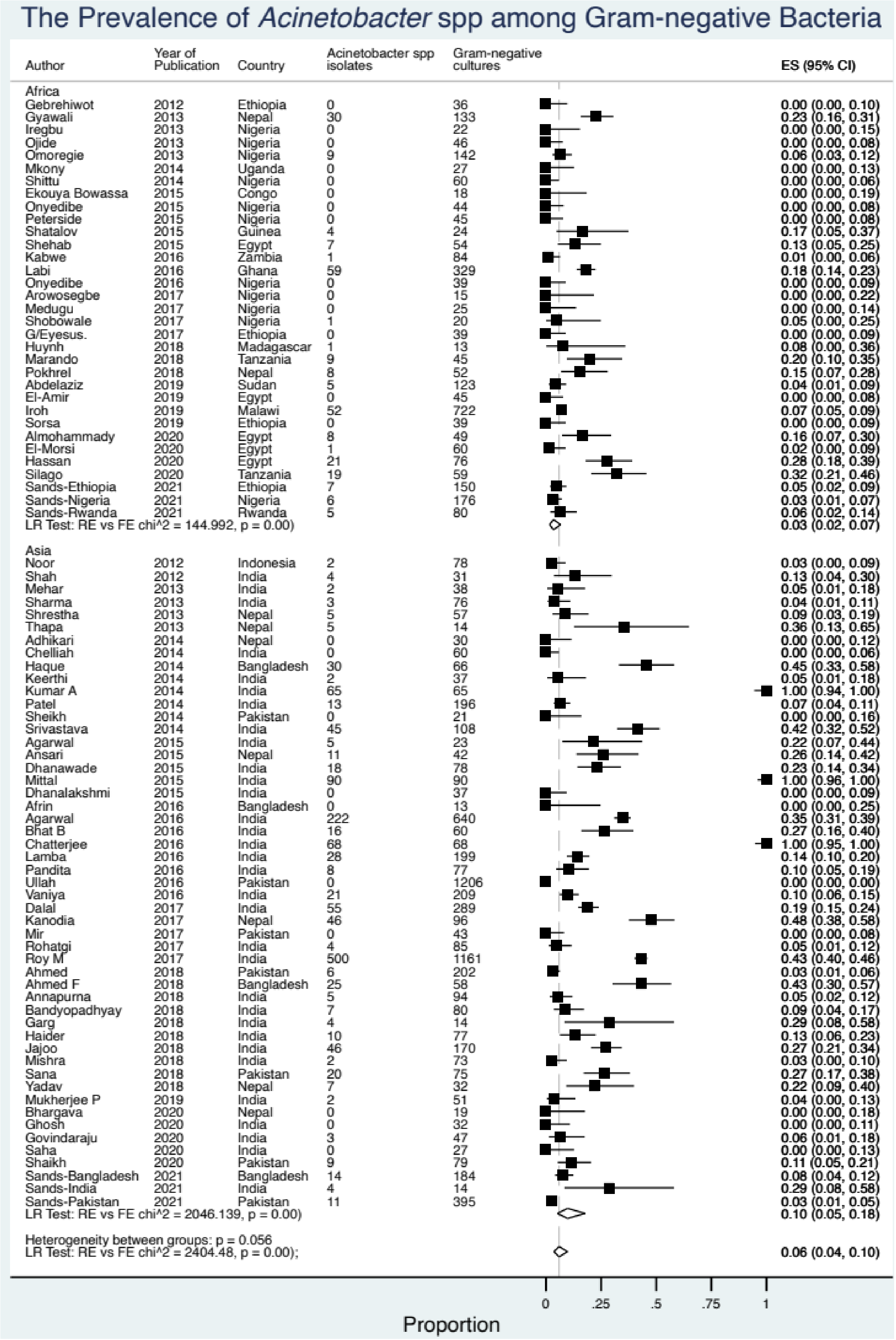
The Prevalence of Acinetobacter spp among Gram-negative Bacteria
Author | Year of Publication | Country | Acinetobacter spp isolates | Gram-negative cultures | ES (95% CI)
Africa
Gebrehiwot 2012 Ethiopia 0 36 0.00 (0.00, 0.10)
Gyawali 2013 Nepal 30 133 0.23 (0.16, 0.31)
Iregbu 2013 Nigeria 0 22 0.00 (0.00, 0.15)
Ojide 2013 Nigeria 0 46 0.00 (0.00, 0.08)
Omoregie 2013 Nigeria 9 142 0.06 (0.03, 0.12)
Mkony 2014 Uganda 0 27 0.00 (0.00, 0.13)
Shittu 2014 Nigeria 0 60 0.00 (0.00, 0.06)
Ekouya Bowassa 2015 Congo 0 18 0.00 (0.00, 0.19)
Onyedibe 2015 Nigeria 0 44 0.00 (0.00, 0.08)
Peterside 2015 Nigeria 0 45 0.00 (0.00, 0.08)
Shatalov 2015 Guinea 4 24 0.17 (0.05, 0.37)
Shehab 2015 Egypt 7 54 0.13 (0.05, 0.25)
Kabwe 2016 Zambia 1 84 0.01 (0.00, 0.06)
Labi 2016 Ghana 59 329 0.18 (0.14, 0.23)
Onyedibe 2016 Nigeria 0 39 0.00 (0.00, 0.09)
Arowosegbe 2017 Nigeria 0 15 0.00 (0.00, 0.22)
Medugu 2017 Nigeria 0 25 0.00 (0.00, 0.14)
Shobowale 2017 Nigeria 1 20 0.05 (0.00, 0.25)
G/Eyesus. 2017 Ethiopia 0 39 0.00 (0.00, 0.09)
Huynh 2018 Madagascar 1 13 0.08 (0.00, 0.36)
Marando 2018 Tanzania 9 45 0.20 (0.10, 0.35)
Pokhrel 2018 Nepal 8 52 0.15 (0.07, 0.28)
Abdelaziz 2019 Sudan 5 123 0.04 (0.01, 0.09)
El-Amir 2019 Egypt 0 45 0.00 (0.00, 0.08)
Iroh 2019 Malawi 52 722 0.07 (0.05, 0.09)
Sorsa 2019 Ethiopia 0 39 0.00 (0.00, 0.09)
Almohammady 2020 Egypt 8 49 0.16 (0.07, 0.30)
El-Morsi 2020 Egypt 1 60 0.02 (0.00, 0.09)
Hassan 2020 Egypt 21 76 0.28 (0.18, 0.39)
Silago 2020 Tanzania 19 59 0.32 (0.21, 0.46)
Sands-Ethiopia 2021 Ethiopia 7 150 0.05 (0.02, 0.09)
Sands-Nigeria 2021 Nigeria 6 176 0.03 (0.01, 0.07)
Sands-Rwanda 2021 Rwanda 5 80 0.06 (0.02, 0.14)
LR Test: RE vs FE chi^2 = 144.992, p = 0.00) 0.03 (0.02, 0.07)
Asia
Noor 2012 Indonesia 2 78 0.03 (0.00, 0.09)
Shah 2012 India 4 31 0.13 (0.04, 0.30)
Mehar 2013 India 2 38 0.05 (0.01, 0.18)
Sharma 2013 India 3 76 0.04 (0.01, 0.11)
Shrestha 2013 Nepal 5 57 0.09 (0.03, 0.19)
Thapa 2013 Nepal 5 14 0.36 (0.13, 0.65)
Adhikari 2014 Nepal 0 30 0.00 (0.00, 0.12)
Chelliah 2014 India 0 60 0.00 (0.00, 0.06)
Haque 2014 Bangladesh 30 66 0.45 (0.33, 0.58)
Keerthi 2014 India 2 37 0.05 (0.01, 0.18)
Kumar A 2014 India 65 65 1.00 (0.94, 1.00)
Patel 2014 India 13 196 0.07 (0.04, 0.11)
Sheikh 2014 Pakistan 0 21 0.00 (0.00, 0.16)
Srivastava 2014 India 45 108 0.42 (0.32, 0.52)
Agarwal 2015 India 5 23 0.22 (0.07, 0.44)
Ansari 2015 Nepal 11 42 0.26 (0.14, 0.42)
Dhanawade 2015 India 18 78 0.23 (0.14, 0.34)
Mittal 2015 India 90 90 1.00 (0.96, 1.00)
Dhanalakshmi 2015 India 0 37 0.00 (0.00, 0.09)
Afrin 2016 Bangladesh 0 13 0.00 (0.00, 0.25)
Agarwal 2016 India 222 640 0.35 (0.31, 0.39)
Bhat B 2016 India 16 60 0.27 (0.16, 0.40)
Chatterjee 2016 India 68 68 1.00 (0.95, 1.00)
Lamba 2016 India 28 199 0.14 (0.10, 0.20)
Pandita 2016 India 8 77 0.10 (0.05, 0.19)
Ullah 2016 Pakistan 0 1206 0.00 (0.00, 0.00)
Vaniya 2016 India 21 209 0.10 (0.06, 0.15)
Dalal 2017 India 55 289 0.19 (0.15, 0.24)
Kanodia 2017 Nepal 46 96 0.48 (0.38, 0.58)
Mir 2017 Pakistan 0 43 0.00 (0.00, 0.08)
Rohatgi 2017 India 4 85 0.05 (0.01, 0.12)
Roy M 2017 India 500 1161 0.43 (0.40, 0.46)
Ahmed 2018 Pakistan 6 202 0.03 (0.01, 0.06)
Ahmed F 2018 Bangladesh 25 58 0.43 (0.30, 0.57)
Annapurna 2018 India 5 94 0.05 (0.02, 0.12)
Bandyopadhyay 2018 India 7 80 0.09 (0.04, 0.17)
Garg 2018 India 4 14 0.29 (0.08, 0.58)
Haider 2018 India 10 77 0.13 (0.06, 0.23)
Jajoo 2018 India 46 170 0.27 (0.21, 0.34)
Mishra 2018 India 2 73 0.03 (0.00, 0.10)
Sana 2018 Pakistan 20 75 0.27 (0.17, 0.38)
Yadav 2018 Nepal 7 32 0.22 (0.09, 0.40)
Mukherjee P 2019 India 2 51 0.04 (0.00, 0.13)
Bhargava 2020 Nepal 0 19 0.00 (0.00, 0.18)
Ghosh 2020 India 0 32 0.00 (0.00, 0.11)
Govindaraju 2020 India 3 47 0.06 (0.01, 0.18)
Saha 2020 India 0 27 0.00 (0.00, 0.13)
Shaikh 2020 Pakistan 9 79 0.11 (0.05, 0.21)
Sands-Bangladesh 2021 Bangladesh 14 184 0.08 (0.04, 0.12)
Sands-India 2021 India 4 14 0.29 (0.08, 0.58)
Sands-Pakistan 2021 Pakistan 11 395 0.03 (0.01, 0.05)
LR Test: RE vs FE chi^2 = 2046.139, p = 0.00) 0.10 (0.05, 0.18)
Heterogeneity between groups: p = 0.056
LR Test: RE vs FE chi^2 = 2404.48, p = 0.00); 0.06 (0.04, 0.10)
0
.25
.5
.75
1
Proportion
